# Identification and evolution analysis of the JAZ gene family in maize

**DOI:** 10.1186/s12864-021-07522-4

**Published:** 2021-04-10

**Authors:** Yang Han, Dawn Luthe

**Affiliations:** The Pennsylvania State University, Plant Science, University Park, PA USA

**Keywords:** Maize, Insect resistance, Jasmonate-ZIM domain, Phylogenetic analysis, Selection

## Abstract

**Background:**

Jasmonates (JAs) are important for plants to coordinate growth, reproduction, and defense responses. In JA signaling, jasmonate ZIM-domain (JAZ) proteins serve as master regulators at the initial stage of herbivores attacks. Although discovered in many plant species, little in-depth characterization of JAZ gene expression has been reported in the agronomically important crop, maize (*Zea mays* L.).

**Results:**

In this study 16 JAZ genes from the maize genome were identified and classified. Phylogenetic analyses were performed from maize, rice, sorghum, Brachypodium, and Arabidopsis using deduced protein sequences, total six clades were proposed and conservation was observed in each group, such as similar gene exon/intron structures. Synteny analysis across four monocots indicated these JAZ gene families had a common ancestor, and duplication events in maize genome may drive the expansion of JAZ gene family, including genome-wide duplication (GWD), transposon, and/or tandem duplication. Strong purifying selection acted on all JAZ genes except those in group 4, which were under neutral selection. Further, we cloned three paralogous JAZ gene pairs from two maize inbreds differing in JA levels and insect resistance, and gene polymorphisms were observed between two inbreds.

**Conclusions:**

Here we analyzed the composition and evolution of JAZ genes in maize with three other monocot plants. Extensive phylogenetic and synteny analysis revealed the expansion and selection fate of maize JAZ. This is the first study comparing the difference between two inbreds, and we propose genotype-specific JAZ gene expression might be present in maize plants. Since genetic redundancy in JAZ gene family hampers our understanding of their role in response to specific elicitors, we hope this research could be pertinent to elucidating the defensive responses in plants.

**Supplementary Information:**

The online version contains supplementary material available at 10.1186/s12864-021-07522-4.

## Background

Constantly challenged by a wide spectrum of stressors, plants utilize phytohormones to mediate responses to stress and enhance their survival by partitioning resources between growth, development, and defense [1]. Jasmonates (JAs) has a dominant role in regulating plant gene expression in response to biotic/abiotic stresses, and also aspects of growth and development, such as trichome configuration, root elongation, and senescence [2, 3]. In plants, JA is primarily produced via oxylipin biosynthesis pathway, derived from α-linolenic acid released by membrane lipids. Among the many metabolic conversions of newly synthesized JA, the formation of jasmonoyl-isoleucine (JA-Ile) is critical for plant direct defense upon herbivore damages [4, 5]. JA-Ile activates the binding of co-receptor CORONATINE INSENSITIVE1 (COI1) and transcriptional repressor JASMONATE ZIM domain (JAZ) protein, and tags JAZs for degradation through SCF^COI1^ (SKP1/Cullin/F-box protein complex) E3 ubiquitin-ligase [6]. This degradation releases transcription factor (TF) MYC2 and further enables the induction of JA-responsive genes including JAZ genes [7].

JAZ proteins are from a large protein family called TIFY [8]. TIFY domain (Pfam accession number PF06200) is named after the conserved motif (TIF [F/Y]XG), members from this plant-specific TF family are previously known as ZIM [9]. TIFY proteins could be divided into two classes, with or without the presence of a C2C2-GATA zinc-finger binding domain [10, 11]. Depend on the domain composition, TIFY family is classified into four subfamilies (TIFY, ZML, JAZ, and PPD) [12, 13]. By definition, proteins from TIFY subfamily only contain the TIFY domain. Besides TIFY domain, proteins from ZML subfamily contain an additional CCT and C2C2-GATA domain [12]. Proteins from JAZ subfamilies have TIFY domain, lack GATA and CCT domains, but contain the Jas domain with the characteristic motif SLX2FX2KRX2RX5PY (Pfam accession number PF09425) which is a variant of CCT domain [11, 13]. Like the JAZ proteins, proteins from PPD subfamily also lack GATA and CCT domains, they have an N-terminal PPD domain instead. Proteins of the TIFY, ZML and JAZ subfamilies can be found in both monocot and dicot plants, however, the PPD subfamily is only present in dicots [12].

The core JA signaling model is developed after revealing the JAZ proteins in Arabidopsis [14, 15]. A total of 13 JAZ genes is present in Arabidopsis, all of them (AtJAZ1–12) have the conserved TIFY and Jas domains, except for AtJAZ13 which has divergent domains [16]. Recent transcriptional analysis has shown that transcripts of AtJAZ genes were directly induced in response to insect feeding, wounding, or other developmental and environmental cues [17–19]. As the key negative regulator of JA signaling during the defense response, extended studies focusing on JAZ proteins have been carried out in major dicots species, including Arabidopsis [14, 15, 20], tobacco [21–23], cotton [24] and tomato [25]. However, except for rice [26–29], little is known about the role of JAZ proteins in monocots like maize (*Zea mays* L.) [30, 31]. As one of the most agronomically important crops in the world, significant maize production (6 to 19%) is lost globally as a result of animal pests like insect herbivores [32]. Therefore, enhancing resistance against herbivores by developing more pest-resistant maize plants is always a research focus [33]. Recent studies indicate JA is a major contributor in maize defense, and JA biosynthesis is induced by leaf-feeding herbivores in maize [34, 35]. Interestingly, it’s been noted that Mp708, the insect-resistant maize inbred line [36], has constitutively elevated JA levels even before herbivore feeding and is “genetically” primed to withstand herbivore attack when comparing with Tx601, the insect-susceptible inbred line [35].

Since JAZ proteins have an important role in regulating JA signaling in *Arabidopsis*, we wanted to determine if similar JAZ genes were present in the maize inbreds Mp708 and Tx601, and determine if there were sequence differences in JAZ between these two inbreds that could explain the differences in constitutive JA levels and herbivore resistance. First, we conducted genome-wide searches for JAZ homologs in maize and three other monocots plant databases (rice, sorghum, and *Brachypodium*). The identified JAZ candidates were further classified based on amino acid sequences and domain composition. Phylogenetic trees and syntenic analyses were then generated among four plant species mentioned above. Lastly, three selected JAZ genes (JAZ1a, 1b; JAZ2a, 2b; JAZ3–1a, 3–1b) were cloned, sequenced, and compared from the insect-resistant maize inbred Mp708 and the insect-susceptible inbred Tx601. The results from this study could provide fundamental information for functional analysis of ZmJAZ genes and the JA signaling pathway in maize plants under insect attack.

## Results

### Identification of the JAZ family in the maize genome

**Fig. 1:**
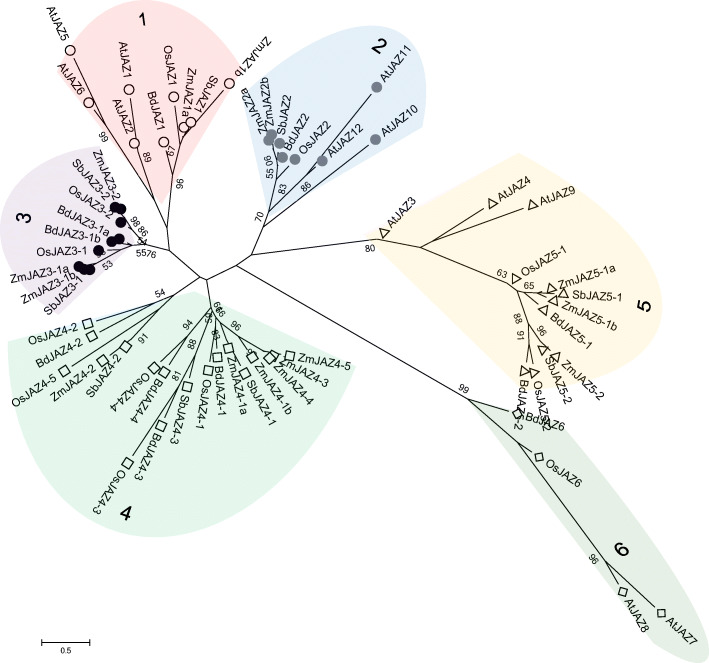
Phylogenetic tree of the JAZ proteins from maize, rice, sorghum, Brachypodium, and Arabidopsis. The tree was constructed using the amino acid sequences by Maximum Likelihood methods with MEGA, the numbers on the branch indicate bootstrap values from 1000 replicates, the cut off value is 50%. Members belonging to the same class were presented with the same label and shaded in color groups (group1, clear circle, red; group 2, grey circle, blue; group 3, black circle, purple; group 4, square, green; group 5, triangle, yellow; group 6, diamond, grey-green). Sources of amino acid sequences are listed in Supplemental Table 1

Thirty-six putative protein sequences were obtained from maize genomes by searching the ZIM [9] domain from GRASSIUM (Grass Regulatory Information Services, https://www.grassius.org) database [37]. Although all these sequences contained the TIFY/ZIM domain, some contained CCT motif and/or C2C2-GATA motif (Group I TIFY protein), thus were predicted as ZML subfamily. Some protein sequences contained only TIFY motifs and were considered belonging to TIFY subfamily. Within the 28 proteins that contained both TIFY domain and Jas motif, two lacked the conserved PY motif at the C-terminal end, two contained incomplete motif, and eight did not have a typical TIFY motif. To identify the most functional JAZ candidates, only the characteristic motifs (“TIFYXG” and “SLX_2_FX_2_KRX_2_RX_5_PY”) were considered in this study (Group II TIFY protein). Other variants including incomplete motifs from the search results were manually eliminated. Overall, 16 members were identified as the ZmJAZ family (Table 1), and these genes were named according to their grouping in phylogenetic (Fig. 1) and synteny analyses (Figs. 3, 4) described below. We also conducted genome-wide searches for JAZ homologs in three other monocot databases and identified 16, 9, and 11 candidate JAZ genes in rice (Supplemental Table 2), sorghum (Supplemental Table 3), and Brachypodium (Supplemental Table 4) genomes, respectively.

**Table 1:** Maize JAZ family

Synonym^**a**^	Protein name	Accession no.	Bin^**b**^	Spl^**c**^	Group	TIFY motif	Jas motif	Loc^**d**^	Org^**e**^	Sta^**f**^	QTL^**g**^
ZmJAZ1a	ZmZIM28	GRMZM2G116614	7.02	2	II	TIFYGG	SLHRFLEKRKDRITAKAPY	N	l	V	SWCB
ZmJAZ1b	ZmZIM13	GRMZM2G005954	2.06	2	II	TIFYGG	SLHRFLEKRKDRITAKAPY	N	l	V	
ZmJAZ2a	ZmZIM34	GRMZM2G143402	10.07	3	II	TIFYGG	SLQRFLEKRRDRVVSKAPY	N	r	V	
ZmJAZ2b	ZmZIM32	GRMZM2G086920	2.02	2	II	TIFYGG	SLQRFLEKRRDRVVSKAPY	N	h,s	R	FAW
ZmJAZ3–1a	ZmZIM23	GRMZM2G089736	7.04	2	II	TIFYGG	SLHRFLEKRKDRLNAKTPY	N	l	V	FAW
ZmJAZ3–1b	ZmZIM12	GRMZM2G101769	2.08	1	II	TIFYGG	SLHRFLEKRKDRLNANAPY	CP	Na	Na	FAW
ZmJAZ3–2	ZmZIM24	GRMZM2G117513	1.04	1	II	TIFYGG	SLRRFLEKRKDRLTAKAPY	N	l	V	
ZmJAZ4–1a	ZmZIM16	GRMZM2G445634	1.02	1	II	TIFYGG	SLQRFLAKRKDRLVERAPY	N	r	V	FAW
ZmJAZ4–1b	ZmZIM4	GRMZM2G036351	9.07	1	II	TIFYGG	SLQRFLAKRKDRLVERAPY	N	r	V	
ZmJAZ4–2	ZmZIM27	GRMZM5G838098	1.02	3	II	TIFYGG	SLKRFLEKRKNRLTAADPY	CP	p	R	FAW
ZmJAZ4–3	ZmZIM9	GRMZM2G338829	6.01	1	II	TIFYGG	SLPWFLTKRKDRLVERAPY	N	Na	Na	
ZmJAZ4–4	ZmZIM19	GRMZM2G382794	1.11	1	II	TIFYGG	SLPWFLAKRKDRLVERAPY	CP	Na	Na	SWCB
ZmJAZ4–5	ZmZIM31	GRMZM2G066020	7.03	1	II	TIFYGG	SLPWFLAKRKDRLVERAPY	G	gs	V	FAW
ZmJAZ5–1a	ZmZIM1	GRMZM2G126507	7.02	2	II	TIFYAG	SLARFLEKRKERVTTAAPY	N	l	V	SWCB
ZmJAZ5–1b	ZmZIM15	GRMZM2G114681	2.06	2	II	TIFYAG	SLARFLEKRKERVTTAAPY	N	a	R,V	
ZmJAZ5–2	ZmZIM35	GRMZM2G151519	4.05	2	II	TIFYNG	SLARFLEKRKERVASVEPY	N	h	R	

^a^ Nomenclature of JAZ subfamily was based on the conserved domains, possible paralogous proteins were grouped togather based on maizesequence.org

^b^ Chromosome number and bin location from maizeGDB

^c^ Number of putative splicing pattern based on maizesequence.org

^d^ Subcellular localization predicted by Protcomp from Softberry: *CP* chloroplast, *G* golgi, *N* nuclear

^e^ Organs with highest expression from maizeGBD: *a* anthers, *gs* germinating seed, *h* husk, *l* leaf, *Na* not available, *p*, pericap, *r*, root, *s* seed, *t* tassel

^f^ Developmental stage with highest expression from maizeGDB: *V* vegetative, *R* reproductive, *Na* not available

^g^ QTLs for insect resistance to FAW and SWCB (Brooks et al., 2007)

Based on information from maizeGDB, the 16 JAZ genes were distributed on seven maize chromosomes: chromosomes 1, 2, and 7 each had four ZmJAZ genes, and chromosomes 4, 6, 9, and 10 each contained one ZmJAZ gene. Because of their possible role in herbivore defense pathway, we were interested in determining if any of the ZmJAZ genes were located in insect-resistance QTLs known for two lepidopteran species, fall armyworm (FAW) and southwestern corn borer (SWCB) [38–40]. As shown in Table 1, six loci were found in regions of FAW QTLs and three were found in regions of SWCB QTLs. In summary, *ZmJAZ1a* and *ZmJAZ5–1a* were located in the SWCB QTL on chromosome 7, bin 0.02, *ZmJAZ2b* and *ZmJAZ3–1b* were located in the FAW QTL on chromosome 2, bin 0.02 and 0.08 respectively, *ZmJAZ3–1a* and *ZmJAZ4–5* were in the FAW QTL on chromosome 7, bin 0.04 and 0.03 respectively, and tandem repeats *ZmJAZ4–1a* and *ZmJAZ4–2* were in the FAW QTL on chromosome 1, bin 0.02.

As a transcription factor, almost all the ZmJAZ proteins had a predicted nuclear localization sequence, but four (*ZmJAZ3–2*, *ZmJAZ4–2*, *ZmJAZ4–4* and *4–5*) had chloroplast or Golgi targeting signals (Table 1). According to the transcriptional analysis by Sekhon [41], the highest expressing organs typically were leaves or roots and different expression patterns for ZmJAZ genes were also listed in Table 1. There was no clear correlation between sequence similarity and gene expression patterns.

### Phylogenetic tree of the JAZ orthologs from maize, rice, sorghum, Brachypodium, and Arabidopsis

To reveal the evolutionary relationship of the JAZ gene family in plants, a phylogenetic tree was created using the deduced protein sequences from maize and orthologous proteins from three monocot genomes used in this study: *Oryza sativa* (12 OsJAZ; Supplemental Table 2), *Sorghum bicolor* (9 SbJAZ; Supplemental Table 3) and *Brachypodium distachyon* (11 BdJAZ; Supplemental Table 4). Besides, 12 JAZ genes from *Arabidopsis thaliana*, a eudicot were also included (Supplemental Table 1). The 60 plant genes analyzed in this study clustered into six orthologous JAZ groups according to the phylogenetic tree (1 to 6, Fig. 1).

Each clade resembles a similar topology order ((ZmJAZa/b, SbJAZ), ZmJAZb/a), (OsJAZ, BdJAZ), AtJAZ) with minor variations. One example was the homologous pair *ZmJAZ2a* and *ZmJAZ2b*, possibly derived from a chromosome duplication event, therefore they were more closely related to each other than *SbJAZ2*. Surprisingly, each monocot species had similar numbers of JAZ proteins from each orthologous group except for group 4. There appeared to be a major expansion in this group both in protein number and sequence divergence. It is noteworthy that members from groups 1, 2, 3, 5 and 6 contained a mixture of protein members from both monocots and dicots plants, however, group 4 appeared to be a monocot-only JAZ clade in this study. Similar results were discovered in other studies, indicating that group 4 might be specific for monocots [42–45]. For example, three *ZmJAZ* genes (4–3, 4–4, 4–5) and one rice gene *OsJAZ4–5* had no orthologous sequences in the other plant genomes.

Results from the phylogenetic analysis showed that all JAZ groups were descended from one ancient origin, and groups 1, 3, 4 and groups 2, 5, 6 were loosely clustered together, indicating a large evolutionary distance between these two groups. Compared with previous analysis of Arabidopsis JAZ proteins, results in this study corresponded to the proposed subclades of AtJAZ proteins [3]. Thanks to the information provided in maize genome database, JAZ genes from the same species in groups 1, 2, and 3 were paralogous, while genes in JAZ groups 4, 5 and 6 were not paralogous with each other. As stated previously, many homologous sequences were not included in this study since they had either incomplete or major changes in one or both of the conserved TIFY and Jas motif. For this reason, group 6 that contains homologous sequences only from rice, Brachypodium, and Arabidopsis, since one homologous sequence in maize (AC187560.5_FGT003) and one in sorghum (Sb02g003130) were manually eliminated.

### Sequence comparison and structure analysis of the maize JAZ genes

**Fig. 2:**
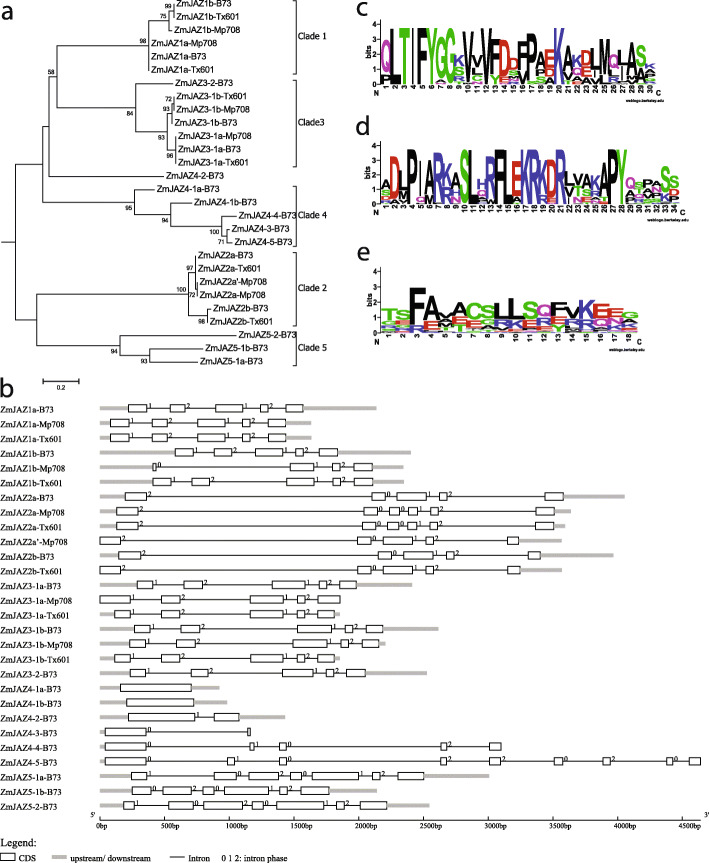
Bioinformatic analysis of the *ZmJAZ* family. **a** Phylogenetic tree of *ZmJAZ* constructed from the deduced amino acid sequence from B73, Mp708, and Tx601. The tree was constructed by Maximum Likelihood methods with MEGA. Numbers on the branch indicate bootstrap values from 1000 replicates. **b** Exon/intron structure of the corresponding *ZmJAZ* gene generated by GSDS. Intron phase numbers are indications of the intron position within a codon: 0, intron not located within a codon (or located between two codons); 1, located between the first and second bases of a codon; 2, located between the second and third bases of a codon. **c** Characterization of core motifs in maize JAZ proteins. Sequences logo of the **b** TIFY motif, **d** Jas motif which contains the conserved PY at the C-terminal end, and **e** CMID motif at the N-terminal end are presented

To gain more insight into the divergence of the 16 maize JAZ genes, a phylogenetic tree was generated using the deduced protein sequences identified in this study (Fig. 2a). JAZ protein families were found in five clades, and members with similar sequences tended to cluster together. ZmJAZ proteins from phylogenetic groups 1, 3, 4 were more closely related compared to groups 2 and 5, and this topology was in line with the phylogenetic tree in Fig. 1, which used JAZ sequences from all five plant species.

Exon/intron structures of the maize JAZ gene family were compared to examine their evolutionary lineages (Fig. 2b). The results showed that ZmJAZ genes with close phylogenetic relationships contained similar exon-intron patterns, including the number of exons, exon length, intron phases, and splicing patterns (Table 1). As shown in Fig. 2b, groups 1, 2, and 3 had five to six exons, group 4 had one to two exons, and group 5 had six to seven exons. However, since exon loss/gain and sequence polymorphisms were identified in the ZmJAZ genes, there is likely functional diversity in the gene family as well. JAZ gene structures in rice (Supplemental Fig. 1), sorghum (Supplemental Fig. 2), and Brachypodium (Supplemental Fig. 3) were also examined. Again, it was striking that members from the same phylogenetic group also shared the identical exon-intron structure among the listed monocot species.

Although the gene sequences among the ZmJAZ family were fairly diverse, two characteristic domains were retained due to their importance for protein-protein interactions: TIFY/ZIM domain was crucial for interactions of JAZ with other transcriptional regulators (i.e. NIJIA, TPL), and Jas domain was important for interactions with bHLH transcription factor (i.e. MYC2) and COI1-mediated protein degradation responding to JA-Ile [8, 17, 46–50]. Particularly in Jas domain, studies revealed a degron sequence LPIAR(R/K) from the N-terminal and the consensus sequences RX5PY from the C-terminal; the former sequence was important for COI1/JA/JAZ complex formation and the latter one served as a nuclear localization signal (NLS) [12, 45, 51]. The phylogenetic relationship was also analyzed (Fig. 2a). To further examine the two conserved domains in ZmJAZ proteins, sequence logos for TIFY and Jas domains (Fig. 2 and Supplemental Fig. 4) were created with WebLogo [52]. The results revealed that both domains (Fig. 2c and d) were highly conserved at multiple amino acid sites. Core domain sequences of the four grass JAZ proteins were listed in Table 1 and Supplemental Tables 2, 3, 4, and the sequences from the same phylogenetic group were found to be highly conserved, with a limited amino acid variation. Besides, another conserved motif cryptic MYC-interaction domain (CMID) (FAX_2_CX_2_LSX_3_K/R) was found near the N-terminus of JAZ proteins (Fig. 2e) using MEME motif search [53]. In Arabidopsis, functional CMIDs have been identified only in *AtJAZ1* and *AtJAZ10* [45]. In maize, CMID domain was more commonly present in JAZ sequences from groups 1, 3 and 4; logo sequences of maize CMID domain were more conserved with *AtJAZ1*. Similar results were found in rice, sorghum, and Brachypodium as well (Supplemental Fig. 5). Interestingly, expression results from a previous study in rice suggested that only proteins containing this motif were induced by both JA and cold stress [42]. The ethylene-response factor amphiphilic repression (EAR) motif (LXLXL) was present at the N-terminus in group 2, this motif was found in NOVEL INTERACTOR OF JAZ (NINJA) and some Arabidopsis JAZ proteins that recruit TOPLESS (TPL) scaffolding proteins to repress jasmonate responses [49].

### Interspecies synteny analysis and expansion patterns of the JAZ genes

**Fig. 3:**
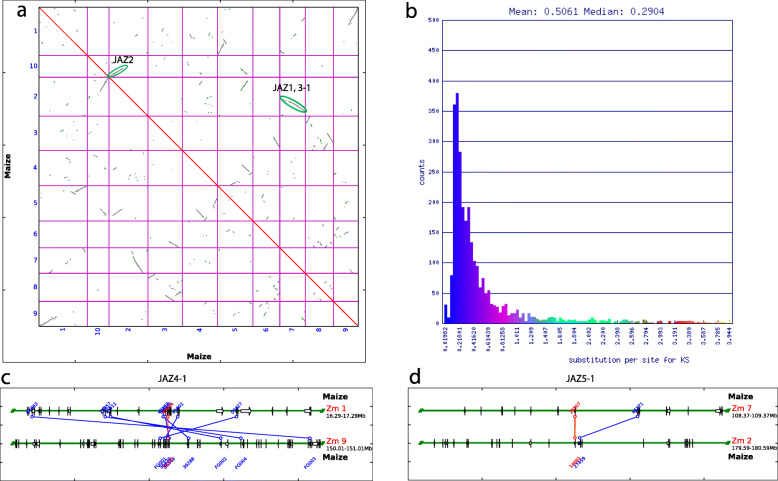
Syntenic comparison of homologous JAZ gene pairs in maize. **a** The synteny dotplot of self-self *Z. mays* genome comparison using SyMAP. Each dot denoted a pair of putative homologous genes that undergone a shared recent WGD event, and syntenic gene pairs were plotted with color based on their Ks values shown in **b**. **b** Histogram of *Ks* values of syntenic gene pairs. The dotplot and Ks histogram were created using CoGe. Three significant syntenic pairs were evident: *ZmJAZ1*, *ZmJAZ3–1*, and *ZmJAZ2* pairs located on the huge syntenic block shared by chromosome 2 and 7, and chromosome 2 and 10, respectively. Smaller syntenic blocks were observed from **c** chromosome 1 and 9 for *ZmJAZ4–1* pairs and **d** chromosome 7 and 2 for *ZmJAZ5–1* pairs generated using PGDD. Syntenic gene pairs were labeled with color lines

Maize chromosomes contain large duplicated regions implying the whole genome duplication (WGD) previously occurred [54]. Such syntenic regions derived from the same ancestral chromosomes could provide some insight into the expansion of the ZmJAZ family. The self-self syntenic dotplot of whole maize genome was presented in Fig. 3, and it provided visual evidence for duplicated regions between maize chromosomes since only the syntenic gene pairs were plotted. On the dotplot, high density of syntenic gene pairs between two chromosomes was represented by color-coded lines with various slopes, based on synonymous substitution rate *Ks* shown in Fig. 3b. When we examined the synteny blocks, three significant syntenic JAZ pairs were identified: *ZmJAZ1a/1b* and *ZmJAZ 3–1a/1b* located on the large syntenic block shared by chromosomes 2 and 7; *ZmJAZ2a/2b* is located on another large syntenic block shared by chromosomes 2 and 10 (Fig. 3a). The other two pairs were observed on syntenic blocks shared by chromosomes 1 and 9 for pair *JAZ4–1a/1b* and chromosome 7 and 2 for pair *JAZ5–1a/1b*, where syntenic gene pairs are labeled with colored lines (Fig. 3c, d).

After WGD, certain duplicated genes were both retained in the genome such as the five JAZ homolog pairs described above. But often, one (or both) copies were lost due to deletion over time [55]. JAZ genes *ZmJAZ3–2, ZmJAZ4–2,* and *ZmJAZ5–2* lost their own duplicated copy, however, they still shared a small syntenic region with *ZmJAZ3–1a*, *ZmJAZ4–1b,* and *ZmJAZ5–1a*, respectively, which was most likely due to an older WGD [56]. *ZmJAZ4–2* and *ZmJAZ4–1a* were defined as a tandem duplication cluster on chromosome 1 since one or no intervening gene was between these two adjacent homologous genes [13]. This was the only tandem duplication event for JAZ genes in the maize chromosomes. There were three genes (*ZmJAZ4–3*, *ZmJAZ4–4*, and *ZmJAZ4–5*) that had no synteny with other genes, nor orthologs in other grass genomes (Fig. 1). The genes in group 4 also had the most exon number variations (one to nine), indicating that loss and gain of exon/intron occurred throughout the evolution of ZmJAZ family. For example, *ZmJAZ4–3*, *ZmJAZ4–4,* and *ZmJAZ4–5* shared a common first exon, but the latter two acquired extra sets of small exons and large introns. By searching in the Plant Genome Duplication Database [57], retrotransposons were found mostly in genes from group 4. Due to the presence of transposon repeats, together with the lack of synteny and corresponding orthologs, *ZmJAZ4–3*, *4–4*, and *4–5* might be the result of transposon duplication. In summary, 13 out of 16 JAZ genes were associated with chromosomal duplications, suggesting these duplication events have contributed to the expansion of maize JAZ gene family.

### Intraspecies synteny analysis of the JAZ family among maize, rice, sorghum, and Brachypodium

**Fig. 4:**
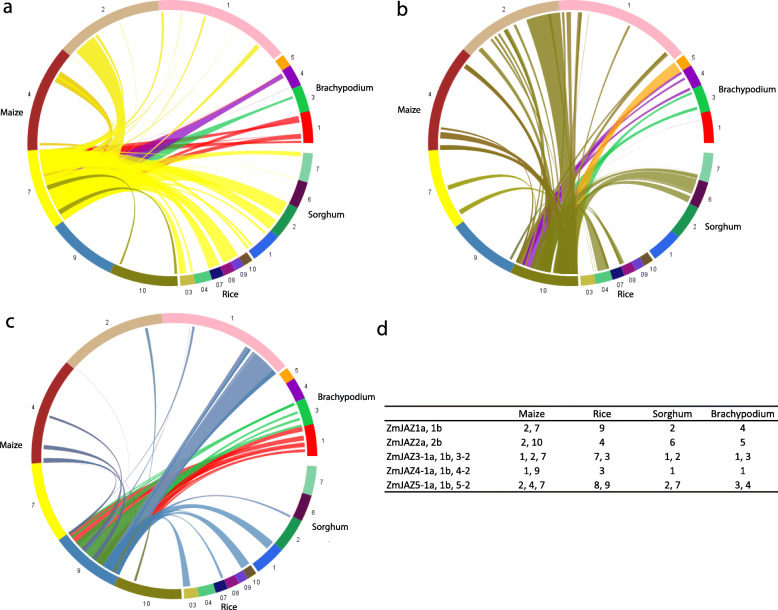
Synteny alignment of the maize, rice, sorghum, and Brachypodium genomes, displayed on the circled scaled map as different color bands with maize genome as reference using SyMAP. Synteny blocks between maize and related grasses were detected and represented with color strips between grass genomes. Chromosome numbers are shown next to the color bar. Major syntenic regions from maize chromosome (1, 2, 4, 7, 9, and 10) where syntenic *ZmJAZ* pairs located were shown in **a** *ZmJAZ1*, *ZmJAZ3*, and *ZmJAZ5*, **b** *ZmJAZ2* and **c** *ZmJAZ4–1a/1b*, and *ZmJAZ4–2*, respectively. A list of synteny blocks from grass genomes (chromosome number) for *ZmJAZ* genes was summarized in **d**

Since all grass species have undergone multiple whole genome duplications (WGD) from a common paleopolyploid ancestry some 70 million years ago (MYA) [58, 59], synteny is evident among different grass families. In this study, four published plant genomes (maize, sorghum, rice, and Brachypodium) were used to represent the grass lineages. To identify orthologous regions among maize and other monocots, we generated several syntenic maps using maize genome as a reference [60] (Fig. 4). Large-scaled synteny blocks containing JAZ orthologs were present across the grass family, which suggests the grass family shared the common ancestor for JAZ genes.

Since the recent WGD in maize, one orthologous region from genomes of rice, sorghum, and Brachypodium had two homologous regions located in maize genome [56]. For example, *ZmJAZ1a/1b* and *5–1a/1b* from maize chromosome (chr) 2 and chr7 aligned with the homologous region in rice chr 9, sorghum chr 2, and Brachypodium chr 4 (Fig. 4a). *ZmJAZ2a/2b* from maize chr 2 and chr 10 were syntenic with rice chr 4, sorghum chr 6, and Brachypodium chr 5 (Fig. 4b). *ZmJAZ4–1a/1b* and *ZmJAZ4–2* from maize chr 1 and chr 9 were syntenic with rice chr 3, sorghum chr 1, and Brachypodium chr 1 (Fig. 4c). A summary of syntenic blocks for ZmJAZ gene was listed in Fig. 4d, including five primary syntenic regions (5 duplicated pairs from Fig. 3: *ZmJAZ1*, *2*, *3–1*, *4–1*, *5–1*) and three secondary syntenic regions for JAZ singleton (*ZmJAZ3–2, 4–2*, and *5–2*) in four plant genomes. It was noteworthy that larger conservation for syntenic JAZ gene pairs was found between the sorghum and maize, which corresponds to the shorter divergence time between the two species (12–18 Mya), although genomic rearrangements were also extensively present in those genomes.

### Strong purifying selection for JAZ genes in maize

Since most of the maize JAZ family was expanded by genome duplications, distances in terms of synonymous (*dS* or *Ks*) and nonsynonymous substitution rates (*dN* or *Ka*) were calculated using a pair-wise comparison of each JAZ orthologous group between maize and the four other plant species (Table 2). Within each maize intra-species comparison (maize-rice, maize-sorghum, maize-Brachypodium, and maize-Arabidopsis), *dS* and *dN* values show homogeneity within most of the orthologous gene groups, however, they were largely different between different intra-species comparisons (ranging from 0.129–0.683 for *dS* and 0.043–0.593 for *dN*). *dS* can often be used to estimate the relative age of homologous sequences [61]. Synonymous distance between maize and the four other plant species can be ranked in the ascending order of Arabidopsis, Brachypodium, rice, maize, and sorghum, which supported the time of divergence based on the phylogenetic lineage. The average *dN* and *dS* values between and within each maize syntenic JAZ gene pair were also estimated and listed in Table 3. *dS* values varied within each syntenic pair (0.181–0.434), with an approximate number 0.1–0.2 for *ZmJAZ2* and *4*, 0.2–0.3 for *ZmJAZ1* and *3*, consistent with the timing of recent WGD event occurred 11–15 MYA ago [54]. The exception was the *ZmJAZ5* gene pair, a higher *dS* (0.434) indicated an older divergence time from each other. Relatively higher *dS* values were also observed between different syntenic pairs, suggesting longer divergence time between each JAZ group.

**Table 2:** Results of distances and codon-based Z tests for purifying selection between maize and other plant species for orthologs JAZ groups

Ortholog	maize-rice		maize-sorghum		maize-brachypodium		maize-Arabidopsis		*dS*-*dN* Stat from test of
clade	*dS*	*dN*	*dS*	*dN*	*dS*	*dN*	*dS*	*dN*	*dS* > *dN* (purifying selection)
JAZ1	0.426 ± 0.042	0.202 ± 0.025	0.143 ± 0.029	0.074 ± 0.014	0.410 ± 0.042	0.231 ± 0.026	0.680 ± 0.039	0.507 ± 0.031	6.117*
JAZ2	0.316 ± 0.044	0.149 ± 0.025	0.129 ± 0.034	0.043 ± 0.014	0.325 ± 0.046	0.126 ± 0.024	0.654 ± 0.047	0.462 ± 0.038	5.250*
JAZ3	0.410 ± 0.041	0.162 ± 0.023	0.285 ± 0.034	0.131 ± 0.019	0.391 ± 0.040	0.189 ± 0.026	0.660 ± 0.041	0.499 ± 0.033	7.947*
JAZ4	0.324 ± 0.058	0.281 ± 0.044	0.245 ± 0.050	0.215 ± 0.038	0.340 ± 0.058	0.271 ± 0.045	n/a	n/a	1.532
JAZ5	0.497 ± 0.038	0.222 ± 0.022	0.379 ± 0.035	0.171 ± 0.018	0.478 ± 0.038	0.234 ± 0.024	0.683 ± 0.034	0.593 ± 0.030	8.495*
JAZ6	n/a	n/a	n/a	n/a	n/a	n/a	n/a	n/a	4.287*
Overall	0.522 ± 0.064	0.308 ± 0.048	0.516 ± 0.063	0.274 ± 0.045	0.533 ± 0.064	0.305 ± 0.047	0.704 ± 0.068	0.364 ± 0.051	7.402*

*Estimations of synonymous and nonsynonymous distance between two species are referred as *dS* and *dN*, respectively. To be considered under purify selection, a *dN/dS* ratio less than 1 (*dS* > *dN*) and a *p*-value for the Z-test below 0.05 were required (*, *P* < 0.05). According to these criteria, almost all JAZ genes were determined to be under purify selection, except for JAZ group 4 which was under neutral selection. Sixty JAZ sequences in total were included in this analysis

**Table 3:** Results of distances and codon-based Z tests for purifying selection between and within JAZ group in maize

	between					within	*dS*-*dN* Stat from test of	*p*-value
	JAZ1	JAZ2	JAZ3	JAZ4	JAZ5	*dN/dS*	*dS* > *dN* (purifying selection)	
JAZ1		0.705	0.536	0.499	0.574	0.076/0.242	3.640*	0.000
JAZ2	0.425		0.639	0.652	0.667	0.048/0.181	2.953*	0.002
JAZ3	0.358	0.464		0.600	0.581	0.140/0.373	4.451*	0.000
JAZ4	0.406	0.415	0.338		0.564	0.165/0.188	0.479	0.316
JAZ5	0.499	0.431	0.411	0.443		0.127/0.434	5.027*	0.000
Overall	–	–	–	–	–	0.361/0.525	4.096*	0.000

*dN/dS values were shown for maize JAZ clades. *dN* and *dS* values were shown separately at lower and upper corner, respectively for between data. To be considered under purify selection, a *dN/dS* ratio less than 1 (*dS>dN*) and a *p*-value for the Z-test below 0.05 were required (*, *P* < 0.05). According to these criteria, almost all JAZ genes were determined to be under purify selection, except for JAZ group 4 which was under neutral selection. 16 ZmJAZ sequences in total were included in this analysis

Comparing orthologs from two species using the *dN*/*dS* ratio could reveal the type of selection pressure acting on the genes: ratio = 1 indicates neutral selection, ratio > 1 indicates positive selection, and radio < 1 indicates purifying selection. Moreover, a codon-based Z-test was also conducted for each JAZ gene using the Nei-Gojobori substitution model/method [62] for purifying (*dN* < *dS*) and the null hypothesis (*dN* = *dS*), and the results were listed in Tables 2 and 3 with *p*-values. After comparing the relative abundance of *dS* and *dN*, we can see almost all group of homologous JAZ genes were under strong purifying selection in the satisfactory zone with p-values less than 0.05. The only exception was genes from group 4, providing a p-value exceeding 0.05 and thus indicating they were under neutral selection. As mentioned before, *ZmJAZ4–1a* and *ZmJAZ4–2* were tandem repeats, and *ZmJAZ4–3*, *4–4*, and *4–5* were transposon repeats without known orthologs with other plant species, the expansion in JAZ group 4 might have happened after the recent WGD since higher *dN*/*dS* ratio suggested a more recent duplications event [63].

### Cloning and characterizing three major homologous JAZ genes from Mp708 and Tx601

This study was undertaken to determine if there were sequence differences in JAZ genes of the insect-resistant genotype Mp708 and the susceptible genotype Tx601 since these two maize inbred lines differed in endogenous JA levels and resistance against Lepidoptera. Based on the genomic identification of JAZ genes from the maize inbred B73, six of the 16 candidate JAZ genes were selected for further analysis: *ZmJAZ1a*/*1b* from group 1, *ZmJAZ2a*/*2b* from group 2, and *ZmJAZ3–1a*/*3–1b* from group 3. There were three reasons why we selected genes from JAZ groups 1, 2 and 3 for testing. First, they had the most conserved sequences when compared across plant JAZ families (Fig. 1), thus there was a higher chance that JA regulatory function was preserved for these genes. Second, they had the highest reported expression in leaves and predicted nucleus locations (Table 1). Third, since *ZmJAZ1* and *ZmJAZ3* were both phylogenetically and functionally closer to each other compared to *ZmJAZ2*, they provided some diversity in the group. Both genomic DNA (gDNA) and cDNA sequences were amplified from maize Mp708 and Tx601 leaves. The resulting amplified fragments were then cloned and sequenced, listed in Table 4.

**Table 4:** Three homologous JAZ genes pairs from maize inbreds Mp708, Tx601

Inbred	Name	Accesion No.	gDNA (bp)	cDNA (bp)^**a**^	protein (aa)	Exon	Intron
Mp708	JAZ1a	MT554628	1632	938	218	5	4
	JAZ1b	MT554629	2345	634	134	4	3
	JAZ2a	MT554630	3639	874	204	6	5
	JAZ2a’	MT554640	3639	943	227	5	4
	JAZ2b	MT554631	3568	x	x	x	x
	JAZ3–1a	MT554632	1856	860	233	5	4
	JAZ3–1b	MT554633	2205	996	237	5	4
Tx601	JAZ1a	MT554634	1633	760	218	5	4
	JAZ1b	MT554635	2342	793	226	5	4
	JAZ2a	MT554636	3594	842	207	6	5
	JAZ2b	MT554637	3569	822	216	5	4
	JAZ3–1a	MT554638	1855	860	233	5	4
	JAZ3–1b	MT554639	2204	857	237	5	4

^a^ For Mp708 and Tx601 inbreds, different splicing pattern was not observed, with the exception of Mp708 JAZ2a’

**Fig. 5:**
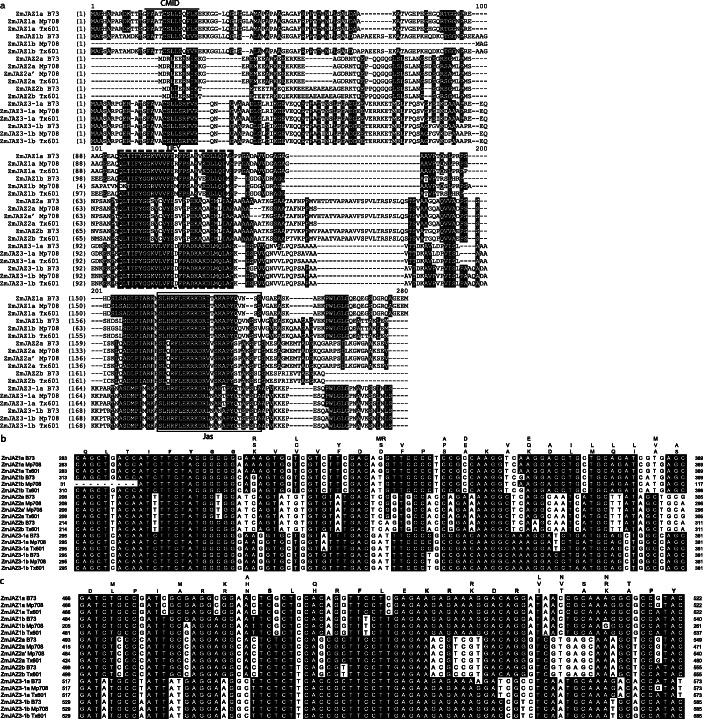
Alignment of homologous ZmJAZ amino acid sequences from group 1, 2, and 3 obtained from three different maize inbreds. **a** The deduced protein sequences of ZmJAZ1, ZmJAZ2, and ZmJAZ3–1 were aligned from maize inbred B73, Mp708, and Tx601 using MEGA. Identical or conserved amino acids are indicated in black backgrounds. The dashes denote spaces required for optimal alignment. Numbers correspond to the amino acid positions. The conserved TIFY domain is labeled with a broken black box, and Jas domain is labeled with a solid black box. Additional conserved CMID motif in the N-terminal is also indicated by consensus sequence FAXXCSLLSXXXK/R. The Sequences were aligned using ClustalW. Comparison of cDNA sequences corresponding to the conserved **b** TIFY domain and **c** Jas domain was also shown. Numbers correspond to the nucleotide position. Black backgrounds indicate identical nucleotides

A comparison of ZmJAZ protein sequences from Table 4 together with paralogs in B73 is shown in Fig. 5a and the conserved domains (TIFY and Jas) were labeled accordingly. Our results revealed that amino acid sequences were quite conserved among homologous pairs for three inbreds, all ZmJAZ pairs exhibited > 60% nucleotide sequence identity, and > 80% peptide sequence identity (Table 5a). When performing a pair-wise comparison between inbreds (Mp708 vs Tx601, Mp708 vs B73, and Tx601 vs B73), there was some degree of polymorphisms present at both nucleotide sequences level (99–100% identity) and amino acid sequences level (94–100% identity) (Fig. 5 and Table 5b). Phylogenetic analysis using the aforementioned protein sequences (Fig. 2a) showed that ZmJAZ sequences from inbreds Mp708, Tx601, and B73 were clustered according to JAZ groups and mini-cluster were formed for each homologous pair. Similar to the previous analysis in Fig. 1, ZmJAZ proteins from groups 1 and 3 were more closely related than JAZ group 2. The protein sequence identity scored highest between group 1 and 3, ranging from 43 to 54%, while the scores were less between the group 1 and 2 and group 2 and 3, ranging from 29 to 44% and 24 to 38%, respectively.

**Table 5:** Pairwise comparisons of sequence similarity between homologous JAZ genes (a) among three maize inbred lines (b)

(a)									
	**Mp708**			**Tx601**			**B73**		
**pair**	**gDNA**	**cDNA**	**protein**	**gDNA**	**cDNA**	**protein**	**gDNA**	**cDNA**	**protein**
JAZ1a, b	68.5	81.4	82.3	70.0	82.9	80.2	61.4	82.9	81.2
JAZ2a, b	73.8	–	–	74.1	89.6	84.9	70.0	89.3	85.1
JAZ3–1a, b	85.0	90.2	87.6	85.0	90.0	87.1	75.7	90.2	86.7
JAZ4–1a, b	–	–	–	–	–	–	83.7	89.4	87.0
JAZ5–1a, b	–	–	–	–	–	–	82.8	89.1	75.8
(b)									
	**Mp708-Tx601**			**Mp708-B73**			**Tx601-B73**		
**Gene**	**gDNA**	**cDNA**	**protein**	**gDNA**	**cDNA**	**protein**	**gDNA**	**cDNA**	**protein**
JAZ1a	99.6	99.2	99.1	99.6	99.8	100.0	99.3	99.1	99.1
JAZ1b	99.3	92.7	94.7	95.9	92.7	94.7	96.6	99.9	100.0
JAZ2a	99.4	99.8	99.5	99.2	99.8	99.5	99.5	100.0	99.0
JAZ2b	100.0	–	–	99.4	–	–	99.4	99.4	99.1
JAZ3–1a	99.6	99.3	99.1	98.5	98.7	98.7	98.9	99.4	99.1
JAZ3–1b	100.0	99.2	100.0	98.6	99.3	99.2	98.5	99.0	99.2

To further explore the variations in conserved TIFY and Jas regions, detailed cDNA sequence alignments were shown in Fig. 5b and c, using the sequences of *ZmJAZ 1a/b*, *Zm*JAZ*2a/b*, and *ZmJAZ3–1a/b* from Mp708, Tx601, and B73. The results indicated the TIFY and Jas domains showed very strong conservation among three inbreds, however, polymorphisms existed at multiple sites. In general, there were more nucleotide substitutions between Mp708 and Tx601, compared with B73. Twelve out of 29, and 16 out of 27 amino acid sites were identical for TIFY and Jas domains, respectively. Polymorphisms were mostly at synonymous sites for each paralogous gene pair due to purifying selection after the recent WGD. On the contrary, polymorphisms were more prevalent at nonsynonymous sites when comparing each inbred, suggesting the possibility of functional divergence for different breeds.

**Fig. 6:**
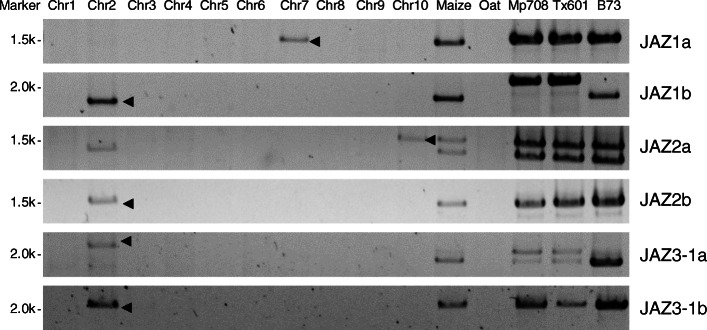
PCR results for the verification of maize JAZ chromosome locations. PCR was performed using specific JAZ primers for gDNA amplification from the oat-maize chromosome addition lines and three maize inbred lines as templates. A total of six homologous JAZ genes were tested and listed on the right panel. The specific PCR bands for each chromosome location were cropped and marked with black arrows. Template gDNAs are indicated at the top: lanes marked Chr1–10 indicate oat-maize addition lines containing maize chromosomes 1–10, respectively; lanes marked maize and oat indicate maize donor and oat background, respectively; lanes marked Mp708, Tx601, and B73 indicate three maize inbred lines used in this study. Agarose gel stained with ethidium bromide was shown above. Full-length gels are presented in Supplemental Fig. 6

To confirm the possible chromosomal location of each cloned ZmJAZ gene, PCR products were generated using gDNA from oat-maize addition lines [64] and together with three maize inbred lines Mp708, Tx601, and B73 (Fig. 6). Chromosome specificity was defined by the presence of an amplified band from the maize gDNA (donor) but absence from oat gDNA [64]. All ZmJAZ genes tested were at the reported locations predicted by the bioinformatics analysis, except for *ZmJAZ3–1a*. This gene was predicted to be located on chromosome 7 but showed a chromosome 2 band on the gel. One possible explanation is the chromosome rearrangement between chromosomes 7 and 2 occurred in the specific maize genomes used to make the oat addition lines, so the location of the gene changed accordingly.

At the sequence level, three paralogs of ZmJAZ gene pairs shown no major variations between Mp708 and Tx601, but differences were present at the transcriptional level (data to be published). Noteworthy, there were several cases where cDNAs of variable lengths were found in Mp708. These differences were clearly visualized in gene structure analysis using cDNA sequences (Fig. 2b). One example was *ZmJAZ1b*, it was significantly shorter in Mp708 than the corresponding genes in Tx601, due to the loss of the first two exons. Another example was *ZmJAZ2a*, there were two cDNA products of *ZmJAZ2a* in Mp708 (*ZmJAZ2a* and *ZmJAZ2a’*) versus only one product in Tx601. Particularly, the two middle exons of *ZmJAZ2a’* in Mp708 were merged but not in others, indicating alternative splicing may have occurred. One more significant difference between Tx601 and Mp708 transcript was that no cDNA product of *ZmJAZ2b* was amplified from Mp708 even when multiple sets of different primers were used. This suggested that *ZmJAZ2b* might not be expressed in Mp708 leaves, although expression was detected in Tx601. Based on the characteristic of three cloned ZmJAZ gene pairs, there were only minor variations at sequence level when comparing the two inbreds; however, more obvious differences were observed at the transcription level, suggest genotype specificity in the expression of maize JAZ genes.

## Discussion

### The phylogenetic relationship of the JAZ genes

It has been shown that JAZ proteins arose after the separation of green algae and land plants, and they are widely present and conserved in all land plant species [9, 12, 65]. A comprehensive study of the JAZ genes in maize and other evolutionary related plant species would provide insights for the origin and evolutionary history of the JAZ family. Our results supported that putative JAZ genes were present in maize and other monocots plants (rice, sorghum, and Brahypodium), which were in line with the previous evolutionary analysis of plant JAZ proteins and topology orders of grass lineages at subfamily level [45, 66]. According to phylogenetic study, six well-supported groups were found representing orthologous JAZ genes in the aforementioned grass family (Fig. 1). Sequences from maize (AC187560.5_FGT003) and sorghum (Sb02g003130) were manually deleted from JAZ group 6 due to loss and/or major changes in the conserved TIFY/Jas domains. It was obvious that members of the same JAZ group were orthologous based on syntenic evidence. The JAZ genes in the same phylogenetic group had remarkable conservation of gene content and exon/intron structure, suggesting that homologous JAZ genes were widely distributed and conserved during the evolution of the grass family. It was also clear that Arabidopsis JAZ proteins were the most distant from maize JAZ proteins, while the sorghum JAZ proteins were the least distant (Fig. 1).

Although all the JAZ groups were from one ancient origin, evolutionarily they were separated into two branches (groups 1, 3, 4 and groups 2, 5, 6) [45]. Our result suggested rice represented the ancestral genome, and the ancestral maize JAZ sequences arose after rice [67]. So we propose a simplified model for maize JAZ sequence evolution based on the evolutionary path of the most conserved domains. ZmJAZ orthologs in groups 2, 5, and 6 diverged independently from the common ancestry. JAZ genes in group 5 separated first, and groups 2 and 6 arose much later in this branch, possibly through recent WGD. Since its early divergence, orthologs in this evolutionary path may originate new functional JAZ proteins. Another independent branch is composed of orthologs in groups 1, 3 and 4, with JAZ genes in group 3 differentiated earlier then followed by separation of group 1 and group 4. For example, in addition to the two highly conserved TIFY and Jas motifs featured in ZmJAZ (Fig. 2c, d), proteins from groups 1, 3, and 4 had another conserved CMID motif (FAX_2_CX_2_LSX_3_K/R) at the N-terminal (Fig. 2e), but proteins from group 2, 5, and 6 did not have this motif (Fig. S5). Another example was EAR motif (LxLxL), which was present at the N-terminal of *ZmJAZ2* from group 2 (also in *AtJAZ 5–8*), but not in other ZmJAZ.

Sequence diversification was also observed between monocot and dicots species, all the JAZ groups mentioned before consisted of gene clusters from both dicots and monocots, except for group 4 which was an exclusive group of monocots JAZ sequences. JAZ orthologs from group 4 possibly originate through WGD from group 3 lineage, and our findings suggested that genes from group 4 evolved specifically in monocots and had some unique features in gene sequences and exon/intron organizations. First, genes from group 4 were under neutral selection rather than purifying selection. Second, most of the members had shorter protein-coding sequences in general and they had a different intron pattern compared with other JAZ genes. For example, most genes had no introns (*OsJAZ4–1*, *OsJAZ4–3*, *OsJAZ4–4*, *SbJAZ4–1*, *SbJAZ4–3*, *BdJAZ4–1*, *BdJAZ4–3*, *BdJAZ4–4*, and *ZmJAZ4–1*), or one to two introns (*OsJAZ4–2*, *OsJAZ4–5*, *SbJAZ4–2*, *BdJAZ4–2*, and *ZmJAZ4–3*), only some genes in maize had multiple introns (*ZmJAZ4–4*, *ZmJAZ4–5*) (Fig. 4b and Supplemental Figs. 1, 2, 3, 4). Third, although most JAZ genes from group 4 could be mapped in syntenic regions among four grass genomes, three maize JAZ genes (*ZmJAZ4–3*, *ZmJAZ 4–4*, and *ZmJAZ 4–5*) and one rice JAZ gene (*OsJAZ4–5*) were not syntenic with any regions. This could be explained by segmental duplication during chromosome rearrangements since these genes showed evidence of transposable elements in the sequence and structure analysis (Fig. 3). The independent evolution of the JAZ genes from group 4 might also generate new functions specifically for monocots plants.

### Expansion pattern of the JAZ genes

Previous literature has stated that maize, rice, sorghum, and Brachypodium experienced multiple rounds of WGDs prior to the modern grass lineage separation [59], so duplication events would give rise to copy numbers of JAZ in each grass genome. The exact number of the JAZ copies were different in each species. In rice, which might represent the ancestral genome [67], it seemed that more copies of JAZ genes were generated during the ancient duplication event. Since less conservation in synteny blocks was observed for duplicated pairs, only small synteny blocks containing genes from JAZ groups 1, 3, and 4 were found in the rice genome. In the rice genome, a majority of the JAZ genes were located in either tandem repeats (*OsJAZ4–1*, *4–2*, *4–3*; *OsJAZ4–4*, *4–5*) or duplicated segmental regions (*OsJAZ3–1*, *3–2*; *OsJAZ5–1*, *5–2*, cluster of *OsJAZ4–1*, *4–2*, *4–3* and *OsJAZ4–4*, *4–5*) [42]. Only two rice JAZ genes (*OsJAZ1* and *OsJAZ2*) were located in the non-duplicated regions. Duplicated events were also present in the *Arabidopsis* genome. Among the 12 JAZ genes in Arabidopsis, four members were the tandem repeats (*AtJAZ1*, *5* and *AtJAZ2*, *6*), and five JAZ pairs were in duplicated regions (*AtJAZ1*, *2*; *AtJAZ3, 4*; *AtJAZ5*, *6*; *AtJAZ7*, *8*, and *AtJAZ11*, *12*). Only *AtJAZ9* and *AtJAZ10* were not from duplication events [9]. Meanwhile, in maize, low *dS* values (0.2–0.4) and low sequence polymorphisms were observed in general for homologous JAZ sequences (Table 1 and Table 3), confirming that they were produced by recent WGD events. The majority of the maize JAZ genes were detected within the syntenic regions of the grass chromosomes (Fig. 4), including 13 ZmJAZ genes: five duplicated pairs (labeled with suffix a and b) were in duplicated chromosome regions including *ZmJAZ1a/b*, *ZmJAZ2a/b*, *ZmJAZ3–1a/b*, *ZmJAZ4–1a/b*, and *ZmJAZ5–1a/b* (Fig. 3), which had five corresponding primary syntenic regions in rice, sorghum, and Brachypodium, respectively; together with three secondary syntenic regions for *ZmJAZ3–2*, *ZmJAZ 4–2*, and *ZmJAZ 5–2* singletons (Fig. 4). There were very limited syntenic gene pairs between maize and Arabidopsis genome due to the long period of divergence, and specifically no syntenic JAZ pairs.

In maize, the latest transposon blooms occurred just a few MYA [68] and transposable elements (95% retrotransposons) comprise about two-thirds of the maize genome [67]. Surprisingly, only members of JAZ genes from group 4 had numerous transposon repeat hits when searched in the Plant Genome Duplication Database [57], while other groups had none or a few (Supplemental Table 5). Besides the duplicated ZmJAZ genes, two genes were tandem repeats (*ZmJAZ4–1a* and *ZmJAZ4–2*), and three members were possible transposon repeats (*ZmJAZ4–3*, *4–4,* and *4–5*). This result provided evidence that the expansion of *ZmJAZ4–3*, *4–4*, and *4–5* might due to transposon insertions in maize, confirm the previous results in sequence and structural analyses (Fig. 1). Similar results were also found in sorghum and Brachypodium (except for group 6), except in rice, all the JAZ genes had many transposon repeats. In conclusion, duplication events, including WGD, tandem duplication, and transposon insertions, contributed to the formation and expansion of JAZ family in plants.

### Selection and functional diversity, the fate of the duplicated JAZ genes

There was a recent WGD event (12–18 Mya ago) in maize after the speciation event of rice and other grass species [56]. Interestingly, there were not twice as many JAZ genes in maize in total number, nor more paralogous gene pairs present between rice and sorghum. Clearly, extensive gene loss and genomic rearrangements occurred in a species-specific pattern after duplication over the long evolutionary history [69]. Because of this, in this study, we could not easily distinguish paralogous pairs because of frequent gene loss and translocation. For example, three singleton JAZ genes lost their duplicated copy such as *ZmJAZ3–2*, *ZmJAZ4–2*, and *ZmJAZ5–2*, however, these genes could still be assigned to the small syntenic regions containing *ZmJAZ3–1a*, *ZmJAZ4–1b*, and *ZmJAZ5–1a*, respectively. So why some duplicated genes were preserved, while others were restored to singletons? Multiple models predict the possible outcomes of duplicated genes after genome duplications, depending extensively on gene features, such as gene context and structural complexity [70]. In the case of the recent WGD duplicates in maize, both copies of the duplicated gene might be retained under strong dosage balance selection [71] as losing one copy likely to cause dosage imbalance. However, the evolutionary force might decrease with time after WGD and one copy of the duplicated gene could be conserved but changes at the amino acid level in the duplicated copy might lead to different fates. Different types of duplication events are under different selective pressures [72]. Recent evidence suggests that higher expressing genes in the population are likely to experience less gene loss than less-frequently expressed genes [56]. For protein-coding sequences, deleterious alleles of highly expressed genes were removed by purifying selection, whereas mutations were accumulated in less frequently expressed genes because they were very likely under neutral or near-neutral selections [56].

Gene duplication provides new materials for selection to act on, thus helping a species adjusting to the rapidly changing environments. The grass family has accumulated a large number of JAZ genes through duplication and transposon insertion, in this study, evidence of purifying selection acting on the putative maize JAZ genes was obtained based on analysis of the *dN*/*dS* values in coding regions, since positive *dS*/*dN* (or low *dN*/*dS*) indicated that there could be purifying selection operating on a gene. The retained genes that were under strong purifying selection may have evolved with little divergence, and their gene functions are likely to be conserved. The only exception was genes from JAZ group 4, which were selectively neutral. Similar results were found in the JAZ family in other grass genomes. We also performed a codon-based Z test of purifying selection for each JAZ ortholog/paralog pairs between maize-rice, maize-sorghum, maize-Brachypodium, and maize-Arabidopsis. This results (Table 2) provided another piece of significant evidence that all but one JAZ group were under purifying selection. One explanation is that members from JAZ group 4 consist of tandem and transposon repeats, which tend to have larger *dN*/*dS* values [73]. It was noteworthy that many of the manually eliminated sequences shared high homology with the genes from JAZ group 4. Although those sequences were not included in this study due to major changes in conserved motifs, this indirectly illustrated that the duplicated genes in group 4 were not under strong selection since many mutations evolved and remained in the population. Meanwhile, members of this group could have developed more precise or new functions during evolution.

JAZ proteins are reported not only having different binding affinities with diversified TF through various protein interaction motifs but also having diverse expression patterns, resulting in a myriad JA-induced response [3]. Multiple studies have shown that although most reported JAZ genes in rice and Arabidopsis are responsive to JA treatment, they have different expression patterns [8, 14, 74, 75]. Changes in the gene expression patterns or protein interactions could be the result of functional divergence [76], for example, some JAZ genes in rice (i.e., OsZIM 14) responded to abiotic stresses like drought [27, 42], whereas others were induced by bacterial pathogens [77]. Here we propose that JAZ proteins from each group might have versatile roles in plant stress responses [3, 78]. One universal response is that members from JAZ groups 1 and 3 were strongly induced by MeJA treatment, wounding, or other biotic stresses such as herbivore or pathogen attacks in both monocots (*B. distachyon*, rice, maize) and dicot species (apple, Arabidopsis, *B. rapa*, grape, rubber, tobacco) [13, 17, 22, 30, 43, 75, 79–81]. Genes from groups 1 and 3 might play a role in plant defense responses and possibly result in growth inhibition. To name a few, the most highly induced JAZ genes from rice (*OsZIM13*) and Arabidopsis (*AtJAZ1*, *2*, *4*, and *5*) were from this large cluster that consisted of JAZ groups 1 and 3. Under cold, salt or drought stresses, members from JAZ group 4 were mostly up-regulated in many monocot plant species, including maize (ZmTIFY4, 26, 28) [30], rice (OsTIFY11a, 11c, and 11d) [28, 42, 82], *B. distachyon* (BdTIFY11a) [43], and bamboo (PeTIFY2, 6, 9, 20, 22) [44]. It appeared that genes from group 4 were largely related to abiotic stress and plant growth regulation. However, it is also not uncommon that JAZ genes from the same group have unique functions and that genes from different groups share certain similarities. For example, Yu et al. [83] found that *AtJAZ7* from JAZ group 6 might be evolved in inhibiting dark-induced senescence and that shading could significantly induce *AtJAZ7* gene expression and protein stability. A similar observation was also found in *AtJAZ10* from JAZ group 2 [83, 84]. While *AtJAZ8*, the closest homolog of *AtJAZ7*, interacted with different TFs [85] thus attenuated JA-dependent response [86] unlike *AtJAZ7* [87].

### Differences between Mp708 and Tx601

JA regulation pathway in plants is very ancient and it has a broad range of biological roles from growth to defense responses. When plants are challenged by herbivore pests or pathogens, they can recognize specific elicitors from the different attacking organism that are required for fine-tuning of outputs through JA signaling pathways [3]. Because maize inbred line Mp708 has elevated constitutive JA levels and greater herbivore resistance than Tx601, we wondered if there were differences in JAZ gene sequences between the two inbreds that could account for these phenotypic difference. Since species-specific functional divergence are widely present in JAZ proteins [45], we speculate that maize JAZ genes from groups 1 and 3 are more likely to be involved in regulating the JA response. Therefore, we picked six representative JAZ genes from three paralogous pairs (*ZmJAZ1a*/*1b*, *ZmJAZ2a*/*2b*, *ZmJAZ3–1a*/*3–1b*) for further investigation. These JAZ genes were cloned from Mp708 and Tx601, using both gDNA and cDNA as templates. Unlike our expectation, we did not identify major sequence differences between Mp 708 and Tx601 in these ZmJAZ genes. Sequence analysis revealed that there was 99–100% nucleotide sequence similarity, and 94–100% deduced amino acid sequence identity between inbred line Mp708, Tx601, and B73 (Table 5b). This result was consistent with previous *dN dS* analysis, that the majority of JAZ genes were under strong purifying selection. If you looked closer at domain level, all of the conserved motifs were present with certain synonymous substitutions at the third codon positions (Fig. 5), except that the conserved N-terminal CMID motif was only present in *ZmJAZ1a/b* and *ZmJAZ3–1a/b* sequences as mentioned previously. However, variations were observed at the transcript level in resistance inbred Mp708, including loss of exons in *ZmJAZ1b* transcript, two splicing products for *ZmJAZ2a* gene, and no transcript detected for *ZmJAZ2b*. The transcriptional differences for each copy of this homologous gene might be the result of the selection of duplicated genes, as previously stated. After duplication daughter genes can become specialized in function resulting in altered spatial or temporal expression pattern at tissue-specific level [88]. This could explain why *ZmJAZ2b* but not *ZmJAZ2a* had no cDNA expression in Mp708 leaves.

Since transcript conservation and divergence were found between maize inbreds Mp708 and Tx601 despite the sequence similarity, we propose that the differences in caterpillar defense responses between inbreds Mp708 and Tx601 were probably not due to the JAZ gene sequences, but might be explained by different expression patterns of the JAZ proteins or post-transcriptional regulation affecting protein stability. Also, further analysis of expression patterns for JAZ genes will provide more information about the diverse role of JAZ proteins in maize in response to herbivore challenges.

## Conclusions

JAZ proteins have been characterized as the primary regulators in JA-signaling pathways activated by various stresses including insect attack [15, 17, 22, 89–91]. However, limited knowledge about this family in maize is available. This study aimed at the genome-wide discovery of JAZ genes which resulted in the identification of 16 JAZs in maize genome. Characterization of these putative maize JAZ genes, together with the systematic analysis of the gene structure, expansion patterns, and evolutionary history in comparison with four other plant species was done as well. Our results indicated homologous JAZ genes were widely distributed and conserved during the evolution of the grass family; genome duplication was proven a major force for gene expansion. This was likely due to strong purifying selection acting on duplicated copies, with the exception of those from group 4, which appears to be a monocots-specific lineage. Weproposed an evolutionary path for JAZ genes in maize, and to the best of our knowledge, the first to compare the gene composition between two maize inbred lines that vary in insect resistance. Results from three paralogous JAZ pairs suggested polymorphisms were present and genotype-specific gene expression patterns were also observed [92]. Due to high genetic redundancy and functional divergence of JAZ genes in nature, we hope this research could enhance our understanding of how plants use JAZs in responding to various environmental stressors.

## Materials and methods

### Data collection

To identify candidate JAZ family members in monocots, the GRASSIUM (Grass Regulatory Information Services, https://www.grassius.org) [37] database was used to search ZIM [9] from four published plant genomes, including maize (*Zea mays*), rice (*Oryza sativa japonica*), sorghum (*Sorghum bicolor*), and Brachypodium (*Brachypodium distachyon*). All homologous sequences contained a predicted ZIM domain with E-values lower than 1.0E-10 were selected and checked in Pfam (pfam.xfam.org) [93]. Previously identified and published JAZ members in Arabidopsis (*Arabidopsis thaliana*) were retrieved from TAIR (https://www.arabidopsis.org) [94], maize genome data were obtained from Gramene using B73 inbred line (https://www.maizesequence.org) [95], rice genome data were from TIGR (http://rice.plantbiology.msu.edu) [96], sorghum and Brachypodium genome data were from Gramene (https://www.gramene.org) [95] (Supplemental Table 1). The search results for each species were then manually selected using the following criteria: a complete TIFY domain (Pfam accession number PF06200) followed by a complete Jas domain (Pfam accession number PF09425, also named as CCT_2 domain) and no other domain(s) present at the C-terminus, like GATA domain [13]. In this study, only the typical “TIFYXG” motif and “SLX_2_FX_2_KRX_2_RX_5_PY” motif were considered and any other variables from the search results were manually eliminated [11, 12].

### Plant material

Seeds from two maize inbred lines (*Zea mays*) were obtained from W. P. Williams (USDA-ARS Corn Host Plant Resistance Research Unit) at Mississippi State University (Mississippi State, MS): Mp708 is resistant and Tx601 is susceptible to fall armyworm (FAW) infestation [36]. After germination, two to four seedlings were sown in each 18 L pots filled by topsoil (Hagertown Loam). Corn plants were raised in the Plant Science greenhouse at The Pennsylvania State University (University Park, PA) till V8- to V9-leaf stage. FAW eggs were also received from USDA-ARS Corn Host Plant Resistance Research Laboratory. After hatching, larvae were reared on the artificial diet [97] until fifth-instar, then three to five FAW larvae were starved for 1 h and placed in the whorls of the V8- to V9-leaf maize plants. After 6 h of infestation, leaf tissues adjacent to the feeding sites were cut immediately, frozen, and stored at − 80 °C until use. Leaves from whorls of undamaged plants were also collected for further use.

### Genomic DNA extraction

Total genomic DNA was isolated from whorls of V8- to V9-leaf stage Mp708 and Tx601 maize leaves, using CTAB (hexadecyltrimethylammonium bromide) method [98]. DNA quantity was examined by NanoDrop Spectrophotometer ND-1000 (Thermo Fisher Scientific Inc., Waltham, MA), and DNA quality was determined by 1% agarose gels.

### RNA extraction and cDNA synthesis

Total RNA from herbivore-fed leaf samples was extracted with TRIzol Reagent (Invitrogen) and then treated with DNase (Progema Corp., Madison, WI) following the standard protocol. RNA quantity was determined by NanoDrop Spectrophotometer ND-1000 (Thermo Fisher Scientific Inc., Waltham, MA). cDNA was then synthesized using ABI high capacity cDNA reverse transcription kit (Foster City, CA), and an aliquot of 1 μg of total RNA and 2.5 μM oligo-dT20 was used in the standard reaction.

### Gene cloning and sequencing

A total of 12 JAZ genes (*ZmJAZ1a*/*1b*; *ZmJAZ2a*/*2b*; *ZmJAZ3–1a*/*3–1b*) were cloned using both cDNA and gDNA from two inbreds Mp708 and Tx601, respectively. To obtain templates for cDNA amplification, maize leaves were fed by FAW larvae for 6 h. Target genes were amplified with *Taq* polymerase (New England Biolabs, Beverly, MA) with 5% DMSO (dimethyl sulfoxide) added to the reaction mix. Primers used in cloning were listed in Supplemental Table 6 and 7, which covers the complete coding regions of maize JAZ candidates. Products of the correct size from PCR amplifications were gel-purified, ligated using the pGEM®-T easy Vectors (cat. No. A1360, Promega), and transformed with competent *E. coli* TOP10 cells (Invitrogen) following the manufacture’s protocol. White colonies were picked after transformation, and at least five clones were selected and sequenced on an Applied Biosystems 3100 DNA sequencer using vector-specific primers T7 (GTAATACGACTCACTATAGGG) and SP6 (GCTATTTAGGTGACACTATAG). The DNA sequences of the ZmJAZ genes were then assembled using SeqMan from DNASTAR (Madison, WI). Pairwise comparison of cDNA and gDNA sequences from each ZmJAZ gene were aligned using the NCBI [99], exons, introns, and URT regions were then identified based on the sequence alignments. All ZmJAZ genes with complete coding regions were successfully amplified, except for *ZmJAZ2*. Due to its high GC- rich context gene nature and longer sequence span, the forward primer of *ZmJAZ2* was located after the translation starting site, so a shorter amplicon was generated: the *ZmJAZ2b* amplicon began at position + 35 downstream of translation start site (cDNA) and + 180 downstream of transcription start site (gDNA) using template sequence from maize genome database.

### PCR with oat-maize addition lines

PCR amplifications were performed for six JAZ genes (ZmJAZ1a, 1b; ZmJAZ2a, 2b; ZmJAZ3–1a, 3–1b) using genomic DNA from oat-maize chromosome addition lines [64]. The maize donor and oat background DNA were used as templates as well as gDNA from three maize inbred lines Mp708, Tx601, and B73. Specific primers in previous cloning steps for ZmJAZ gDNA were used except for *ZmJAZ2a*/*2b*, whereas primers covering partial gDNA sequence (~ 2 kb) were used since full-length *ZmJAZ2* genes were over 4 kb long. The chromosome location of the maize JAZ genes was determined if the predicted size band was present in one of the chromosomes from oat-maize addition lines and maize donor line, but not in the oat background line. The original gel was cropped to show the specific PCR bands for each chromosome locations, which marked with black arrows in Fig. 6. Full-length gels are available in Supplemental Fig. 6. For maize donor lines, chromosomes 1 to 9 were from Senaco 60, and chromosome 10 was from Mo17. For oat background lines, most of the oat lines were Starter 1, with the exception that SunII was used for chromosome 3 and 5, and Gaf Park for chromosome 8. All primers used here were listed in Supplementary Table 8.

### Phylogenetic tree

Phylogenetic analysis for the JAZ family from multiple plant genomes was conducted in MEGA v6 [100]. Multiple JAZ protein amino acid sequences were aligned by MUSCLE using default settings. The resulting alignment was used for phylogenetic analysis. The best substitution model was selected for Maximum likelihood (ML) inference. According to the best substitution model (JTT + G), ML method was then used for phylogenetic tree construction, with 1000 bootstrap resampling.

A separate analysis was carried out for ZmJAZ gene sequences from B73, Mp708, and Tx601 inbred lines also using MEGA. The ZmJAZ coding sequences were aligned by ClustalW, and then similar method was used to generate a phylogenetic tree. The tree was also constructed using ML method (Tamura 3-parameter+G + I) with 1000 bootstrap resamplings.

### Synteny analysis

Chromosome location, ortholog, and paralog information for the ZmJAZ genes were obtained from MaizeGDB (https://www.maizegdb.org/) [40]. Orthologous JAZ genes were also checked in the Rice Orthologous database (http://rice.plantbiology.msu.edu/annotation_pseudo_pog.shtml) for four grass genomes (maize, rice, sorghum, and Brachypodium) [96]. Adjacent homologous JAZ genes locating on the same chromosome, with one or no intervening gene, were considered as tandem duplications in maize chromosomes [13]. Synteny information was obtained from the Plant Genome Duplication Database (https://chibba.agtec.uga.edu/duplication/#Zea_mays) [57]. Each JAZ gene was searched in the above database, and the syntenic blocks within the maize chromosomes containing the examined genes were identified. The synteny dotplot of self-self *Z. mays* genome was generated by SynMap from CoGe [101]. SyMAP [60] was also used to compute and view the syntenic blocks between and within grass genomes. The results were presented in graphic Java display, which could change from circle view to dotplot view, and 2D view.

### Gene structure and domain analysis

The gene structures with exon/intron positions and gene length were generated utilizing the online Gene Structure Display Server (GSDS; http://gsds.gao-lab.org/) [102] for maize JAZ genes. Motif-based sequence analysis for JAZ proteins was searched in the MEME server (https://meme.nbcr.net/meme/cgi-bin/meme.cgi) with the default setting [103]. Visualization of the consensus sequences was created by WebLogo [52].

### *dS, dN* computing and tests of selection

We used JAZ coding sequences to estimate synonymous rate (*dS*, number of synonymous substitutions per synonymous site) and nonsynonymous rate (*dN*, number of nonsynonymous substitutions per nonsynonymous site) using MEGA v6 [104]. The coding sequences were aligned by ClustalW and *dS*, *dN* was computed based on this alignment using the Nei-Gojobori substitution model/method [62]. Positions with at least 95% site coverage were presented, and bootstrap resampling of 1000 was used. Also, codon-based Z-test was performed on each pair of sequences using MEGA v6, which calculated the relative abundance of synonymous and nonsynonymous substitutions. Then the average score for each orthologous group (JAZ1 to 6) was computed. With the calculated Z-test scores and probability (*p*-value less than 0.05 are considered significant at the 5% level), neutral evolution (*dN* = *dS*), positive selection (*dN* > *dS*) or purifying selection (*dN* < *dS*) [62] were tested.

## Supplementary Information

**Additional file 1: Supplemental Table 1.** List of members of JAZ family in this study. **Supplemental Table 2.** Rice JAZ family. **Supplemental Table 3.** Sorghum JAZ family. **Supplemental Table 4.** Brachypodium JAZ family. **Supplemental Table 5.** Search results from plant repeats database. **Supplemental Table 6.** Primers used in JAZ gDNA cloning. **Supplemental Table 7.** Primers used in JAZ cDNA cloning. **Supplemental Table 8.** Primers used in JAZ PCR of oat-maize addition lines

**Additional file 2: Supplemental Figure 1.** Exon/intron structure of the corresponding OsJAZ gene generated by GSDS. Intron phase numbers were indications of the intron position within a codon: 0, intron not located within a codon (or located between two codons); 1, located between the first and second bases of a codon; 2, located between the second and third bases of a codon. **Supplemental Figure 2.** Exon/intron structure of the corresponding SbJAZ gene generated by GSDS. Intron phase numbers were indications of the intron position within a codon: 0, intron not located within a codon (or located between two codons); 1, located between the first and second bases of a codon; 2, located between the second and third bases of a codon. **Supplemental Figure 3.** Exon/intron structure of the corresponding BdJAZ gene generated by GSDS. Intron phase numbers were indications of the intron position within a codon: 0, intron not located within a codon (or located between two codons); 1, located between the first and second bases of a codon; 2, located between the second and third bases of a codon. **Supplemental Figure 4.** Sequences logo of the (a) TIFY domain, (b) Jas domain, and (c) N-terminal CMID domains from four grass JAZ genes created by WebLogo. **Supplemental Figure 5.** Distribution of conserved motifs in JAZ proteins. (a) Conserved motifs from maize JAZ proteins. (b) Conserved motifs from JAZ groups 1, 3, and 4 in maize, rice, sorghum, Brachypodium, and Arabidopsis. The conserved motifs with non-overlapping sites (*p*-value> 0.0001) were shown in colored boxes generated by MEME server. TIFY, Jas, and N-terminal CMID motifs were represented in motif 1, 2, and 3, respectively. **Supplemental Figure 6.** Full-length gels for PCR results with oat-maize addition lines. PCR was performed using specific JAZ primers for gDNA amplification from the oat-maize chromosome addition lines and three maize inbred lines as templates. A total of six homologous JAZ genes (a-f) were tested and labelled on the right panel. The specific PCR bands for each chromosome location were cropped and presented in Fig. 6. Template gDNAs are indicated at the top: lanes marked Chr1–10 indicate oat-maize addition lines containing maize chromosomes 1–10, respectively; lanes marked maize and oat indicate maize donor and oat background, respectively; lanes marked Mp708, Tx601, and B73 indicate three maize inbred lines used in this study. Agarose gel stained with ethidium bromide was shown above

## Data Availability

The datasets generated and/or analyzed during the current study are available in the TreeBASE repository, https://purl.org/phylo/treebase/phylows/study/TB2:S27563.
