# Supplementary material for: Identification and evolution analysis of the JAZ gene family in maize
Source: BMC Genomics. 2021 Apr 10;22:256. doi: 10.1186/s12864-021-07522-4 (PMC8037931; doi:10.1186/s12864-021-07522-4)
Supplement: Supplementary file 2 — Additional file 2: Supplemental Figure 1. Exon/intron structure of the corresponding OsJAZ gene generated by GSDS. Intron phase numbers were indications of the intron position within a codon: 0, intron not located within a codon (or located between two codons); 1, located between the first and second bases of a codon; 2, located between the second and third bases of a codon. Supplemental Figure 2. Exon/intron structure of the corresponding SbJAZ gene generated by GSDS. Intron phase numbers were indications of the intron position within a codon: 0, intron not located within a codon (or located between two codons); 1, located between the first and second bases of a codon; 2, located between the second and third bases of a codon. Supplemental Figure 3. Exon/intron structure of the corresponding BdJAZ gene generated by GSDS. Intron phase numbers were indications of the intron position within a codon: 0, intron not located within a codon (or located between two codons); 1, located between the first and second bases of a codon; 2, located between the second and third bases of a codon. Supplemental Figure 4. Sequences logo of the (a) TIFY domain, (b) Jas domain, and (c) N-terminal CMID domains from four grass JAZ genes created by WebLogo. Supplemental Figure 5. Distribution of conserved motifs in JAZ proteins. (a) Conserved motifs from maize JAZ proteins. (b) Conserved motifs from JAZ groups 1, 3, and 4 in maize, rice, sorghum, Brachypodium, and Arabidopsis. The conserved motifs with non-overlapping sites (p-value> 0.0001) were shown in colored boxes generated by MEME server. TIFY, Jas, and N-terminal CMID motifs were represented in motif 1, 2, and 3, respectively. Supplemental Figure 6. Full-length gels for PCR results with oat-maize addition lines. PCR was performed using specific JAZ primers for gDNA amplification from the oat-maize chromosome addition lines and three maize inbred lines as templates. A total of six homologous JAZ genes (a-f) were tested and labelled on [file 12864_2021_7522_MOESM2_ESM.pdf]

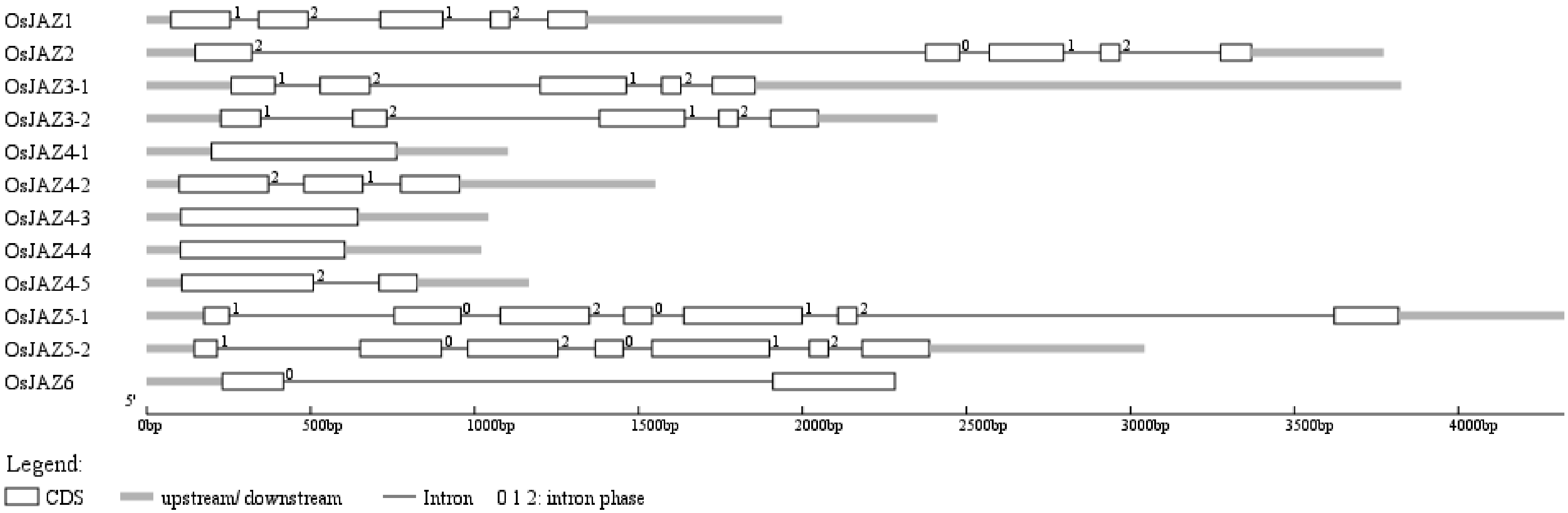

Supplemental Fig. 1 Exon/intron structure of the corresponding OsJAZ gene generated by GSDS. Intron phase numbers were indications of the intron position within a codon: 0, intron not located within a codon (or located between two codons); 1, located between the first and second bases of a codon; 2, located between the second and third bases of a codon

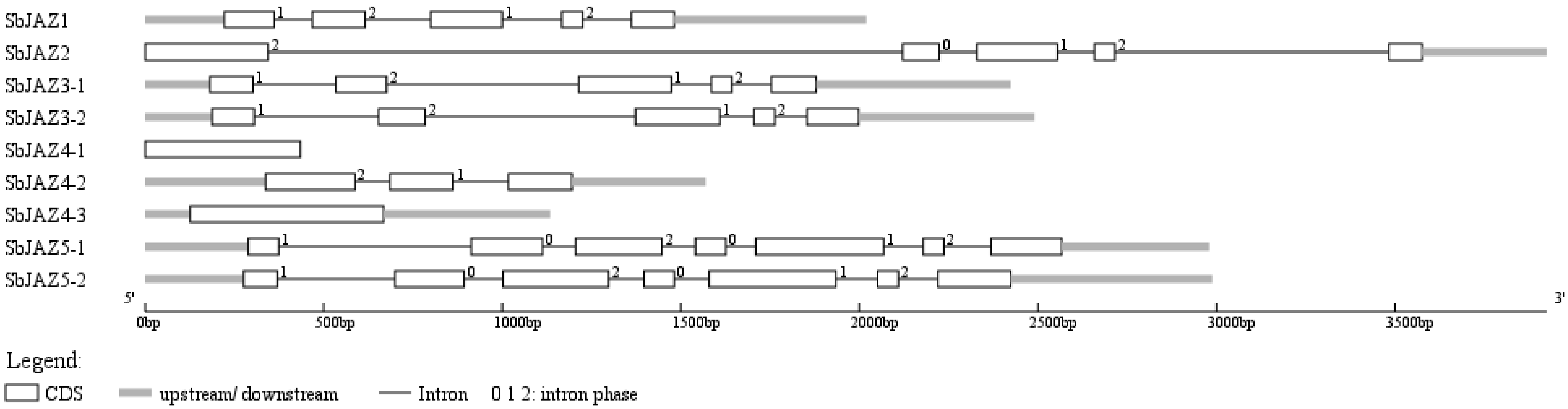

Supplemental Fig. 2 Exon/intron structure of the corresponding SbJAZ gene generated by GSDS. Intron phase numbers were indications of the intron position within a codon: 0, intron not located within a codon (or located between two codons); 1, located between the first and second bases of a codon; 2, located between the second and third bases of a codon

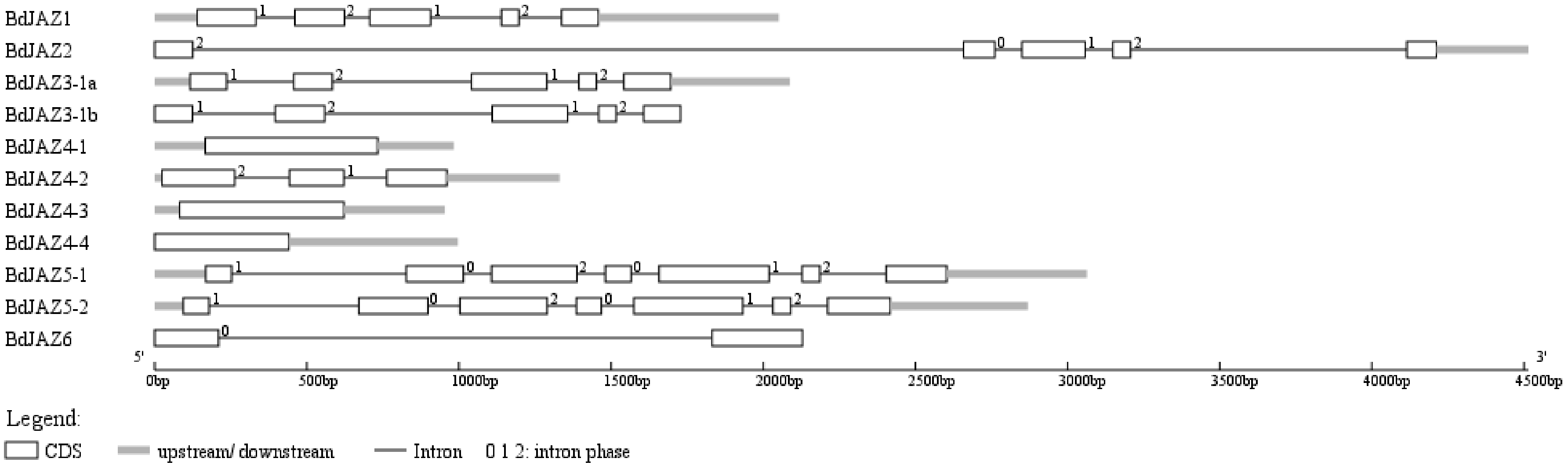

Supplemental Fig. 3 Exon/intron structure of the corresponding BdJAZ gene generated by GSDS. Intron phase numbers were indications of the intron position within a codon: 0, intron not located within a codon (or located between two codons); 1, located between the first and second bases of a codon; 2, located between the second and third bases of a codon

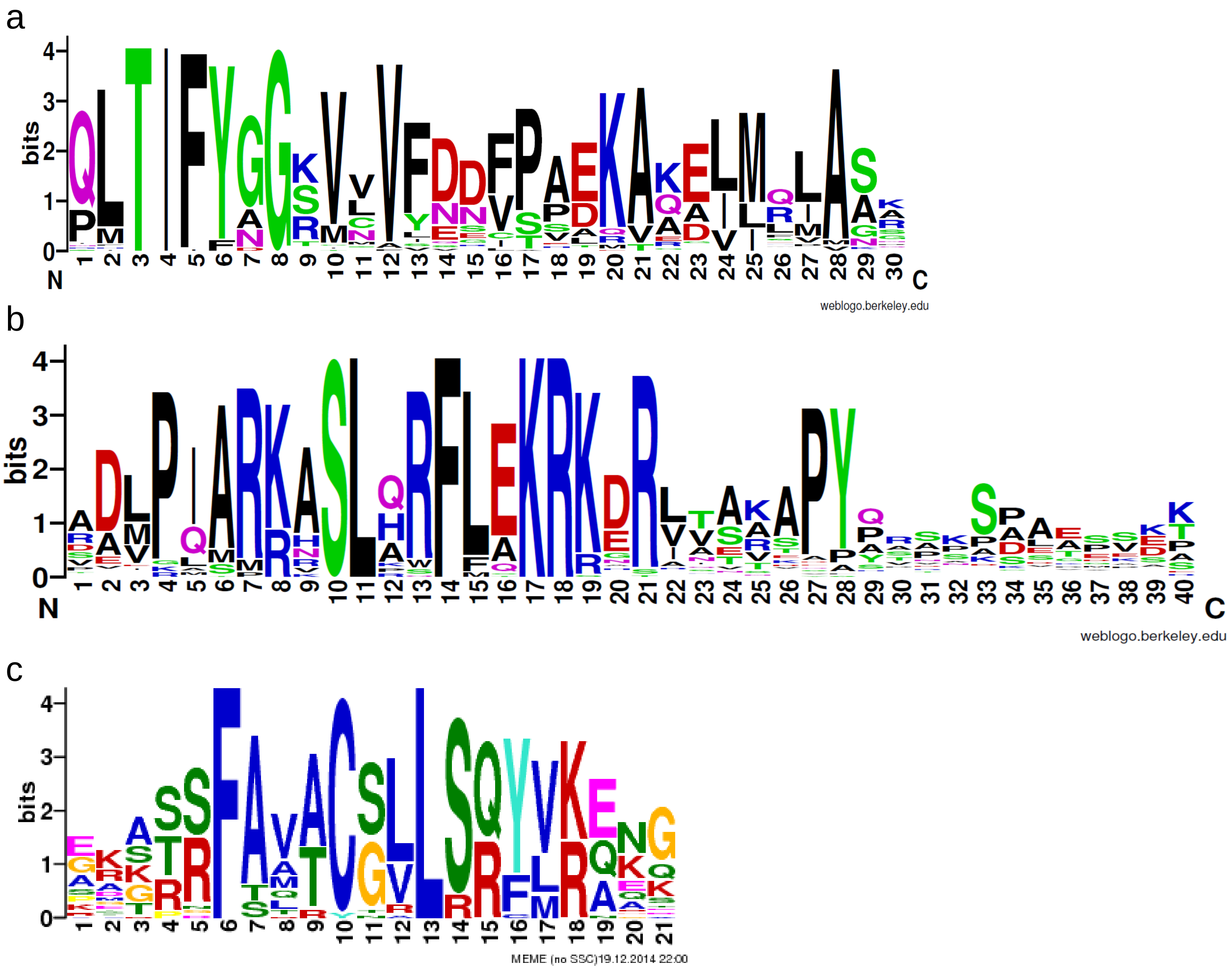

Supplemental Fig. 4 Sequences logo of the (a) TIFY domain, (b) Jas domain, and (c) N-terminal CMID domains from four grass JAZ genes created by WebLogo.

a

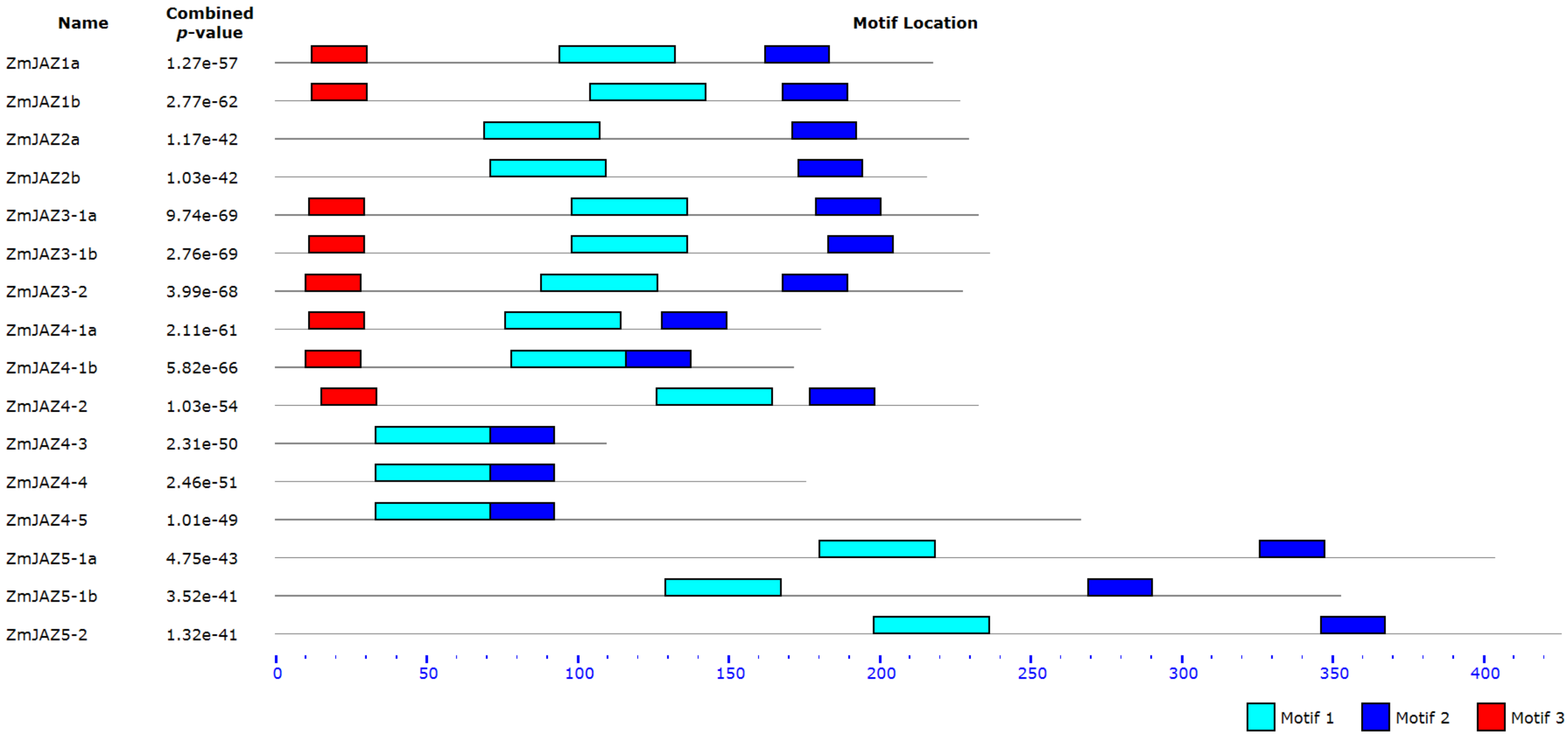
Name
Combined *p*-value
Motif Location
ZmJAZ1a 1.27e-57
ZmJAZ1b 2.77e-62
ZmJAZ2a 1.17e-42
ZmJAZ2b 1.03e-42
ZmJAZ3-1a 9.74e-69
ZmJAZ3-1b 2.76e-69
ZmJAZ3-2 3.99e-68
ZmJAZ4-1a 2.11e-61
ZmJAZ4-1b 5.82e-66
ZmJAZ4-2 1.03e-54
ZmJAZ4-3 2.31e-50
ZmJAZ4-4 2.46e-51
ZmJAZ4-5 1.01e-49
ZmJAZ5-1a 4.75e-43
ZmJAZ5-1b 3.52e-41
ZmJAZ5-2 1.32e-41
0
50
100
150
200
250
300
350
400
Motif 1
Motif 2
Motif 3

b

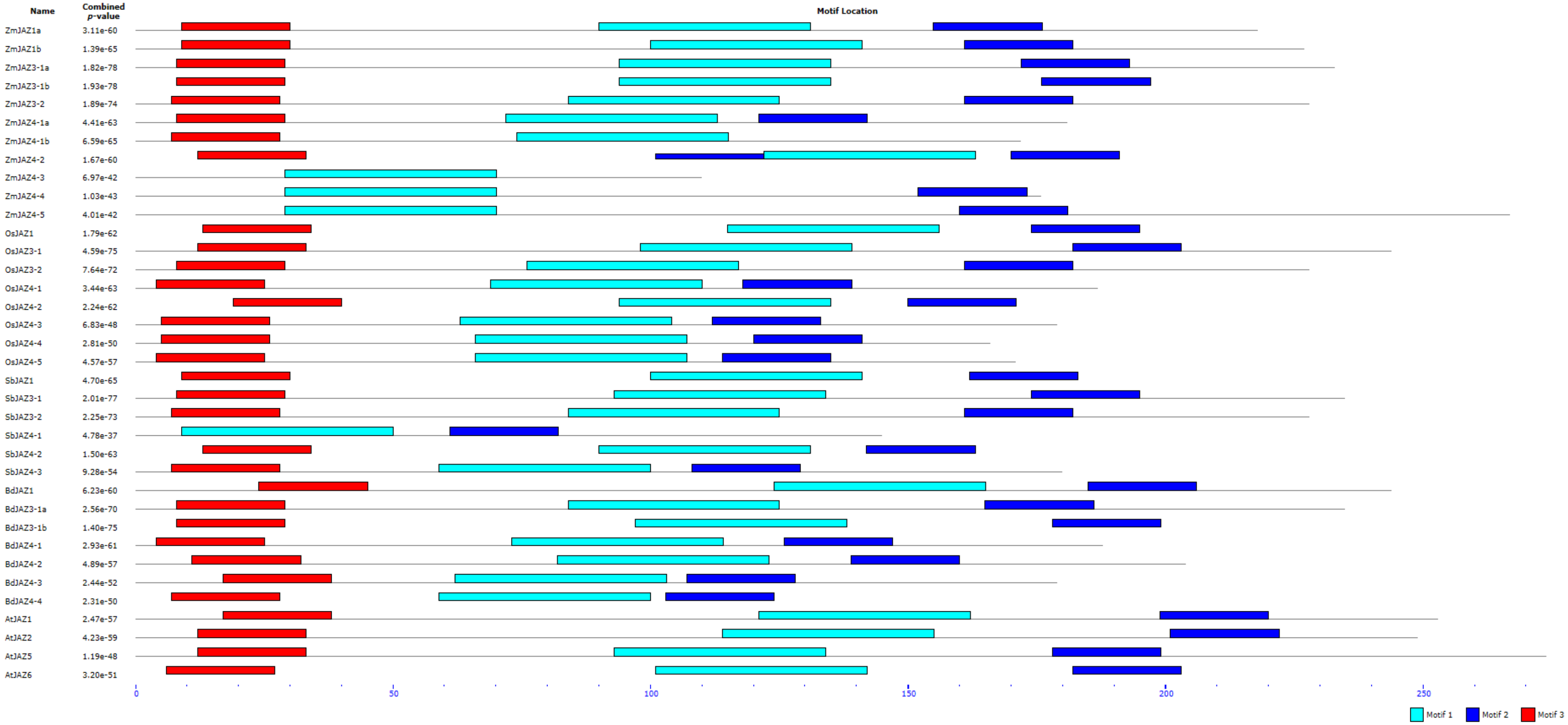

Supplemental Fig. 5 Distribution of conserved motifs in JAZ proteins. (a) Conserved motifs from maize JAZ proteins. (b) Conserved motifs from JAZ groups 1, 3, and 4 in maize, rice, sorghum, Brachypodium, and Arabidopsis. The conserved motifs with non-overlapping sites (*p*-value>0.0001) were shown in colored boxes generated by MEME server. TIFY, Jas, and N-terminal CMID motifs were represented in motif 1, 2, and 3, respectively

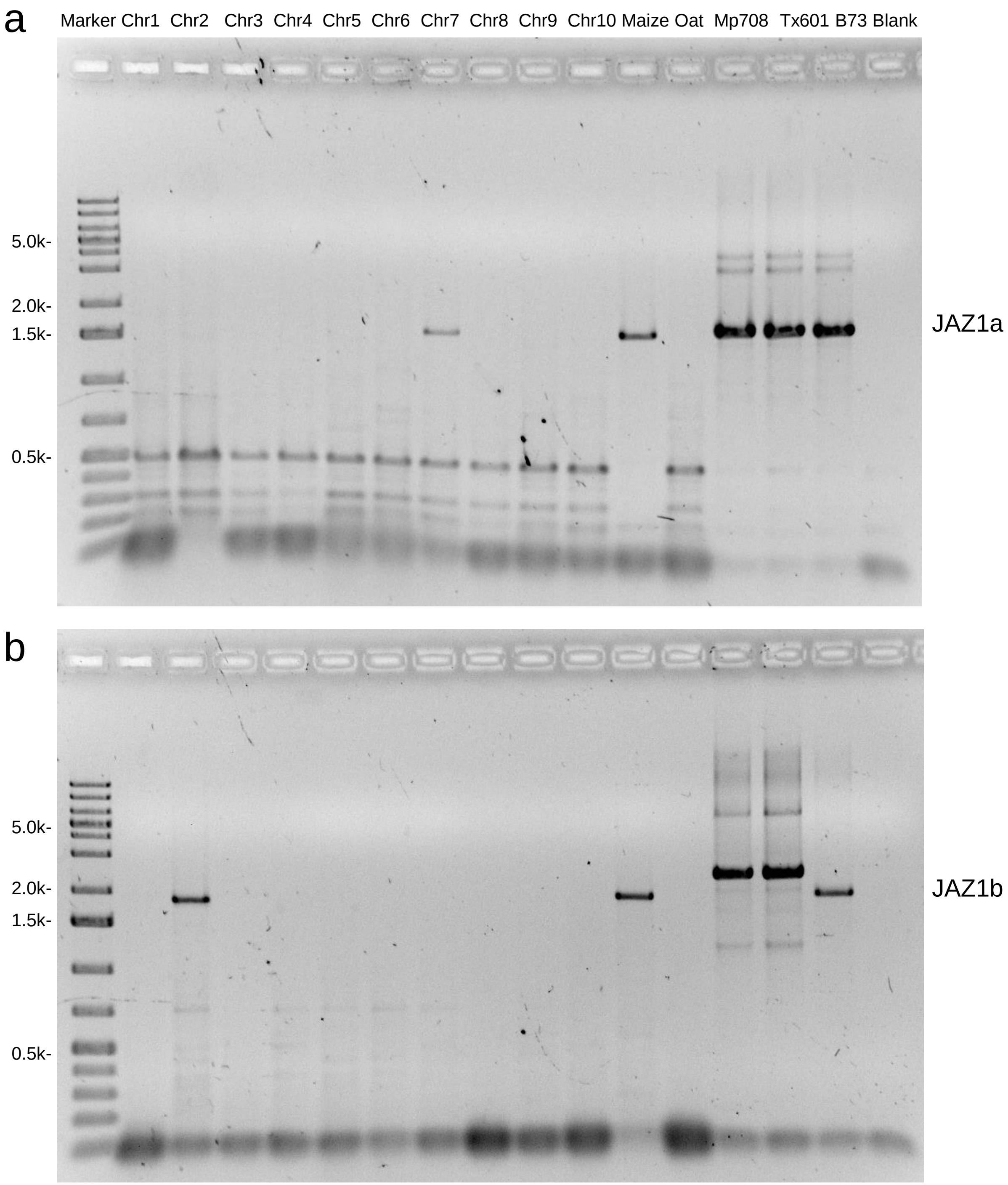
a
Marker Chr1 Chr2 Chr3 Chr4 Chr5 Chr6 Chr7 Chr8 Chr9 Chr10 Maize Oat Mp708 Tx601 B73 Blank
5.0k-
2.0k-
1.5k-
0.5k-
JAZ1a
b
5.0k-
2.0k-
1.5k-
0.5k-
JAZ1b

C

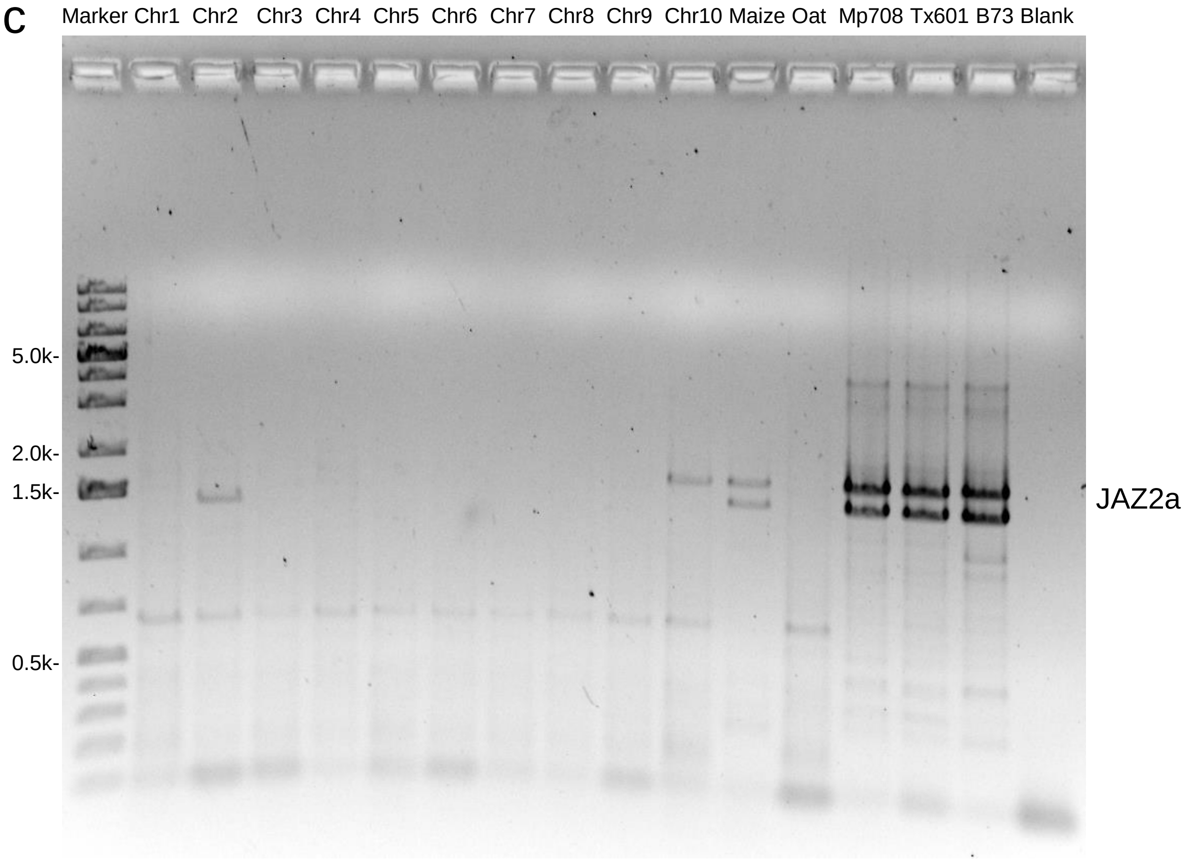

Marker Chr1 Chr2 Chr3 Chr4 Chr5 Chr6 Chr7 Chr8 Chr9 Chr10 Maize Oat Mp708 Tx601 B73 Blank
5.0k-
2.0k-
1.5k-
0.5k-
JAZ2a

d

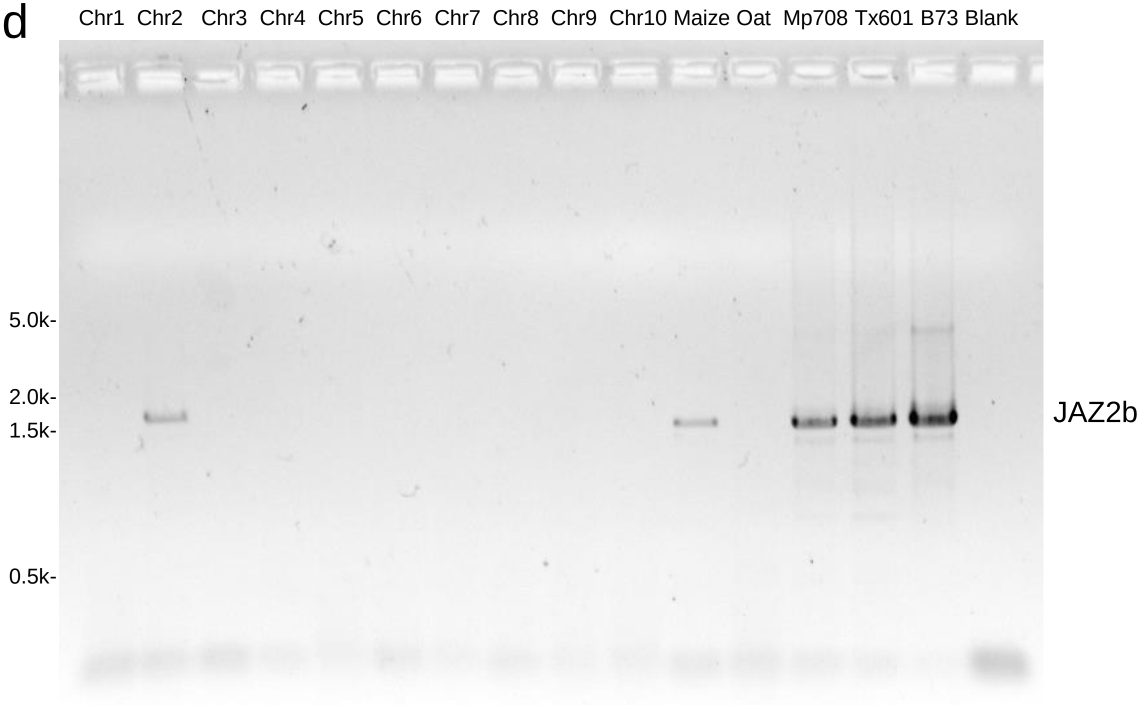
Chr1 Chr2 Chr3 Chr4 Chr5 Chr6 Chr7 Chr8 Chr9 Chr10 Maize Oat Mp708 Tx601 B73 Blank
5.0k-
2.0k-
1.5k-
0.5k-
JAZ2b

e

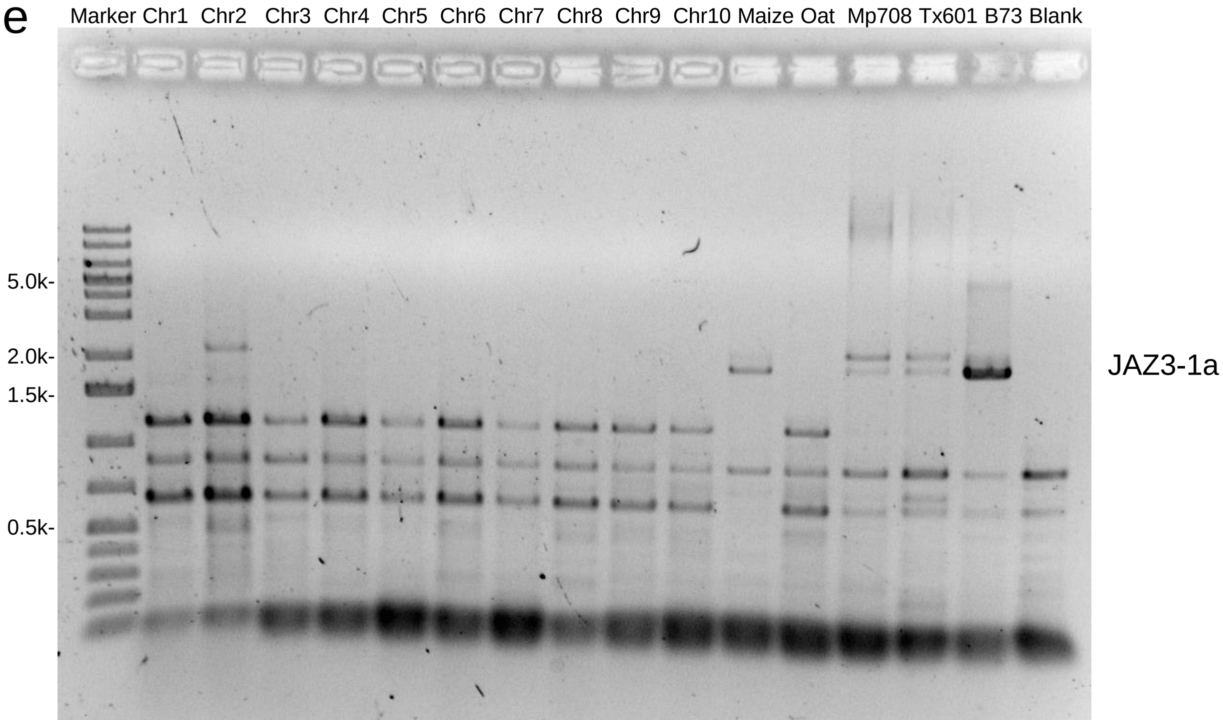
Marker Chr1 Chr2 Chr3 Chr4 Chr5 Chr6 Chr7 Chr8 Chr9 Chr10 Maize Oat Mp708 Tx601 B73 Blank
5.0k-
2.0k-
1.5k-
0.5k-
JAZ3-1a

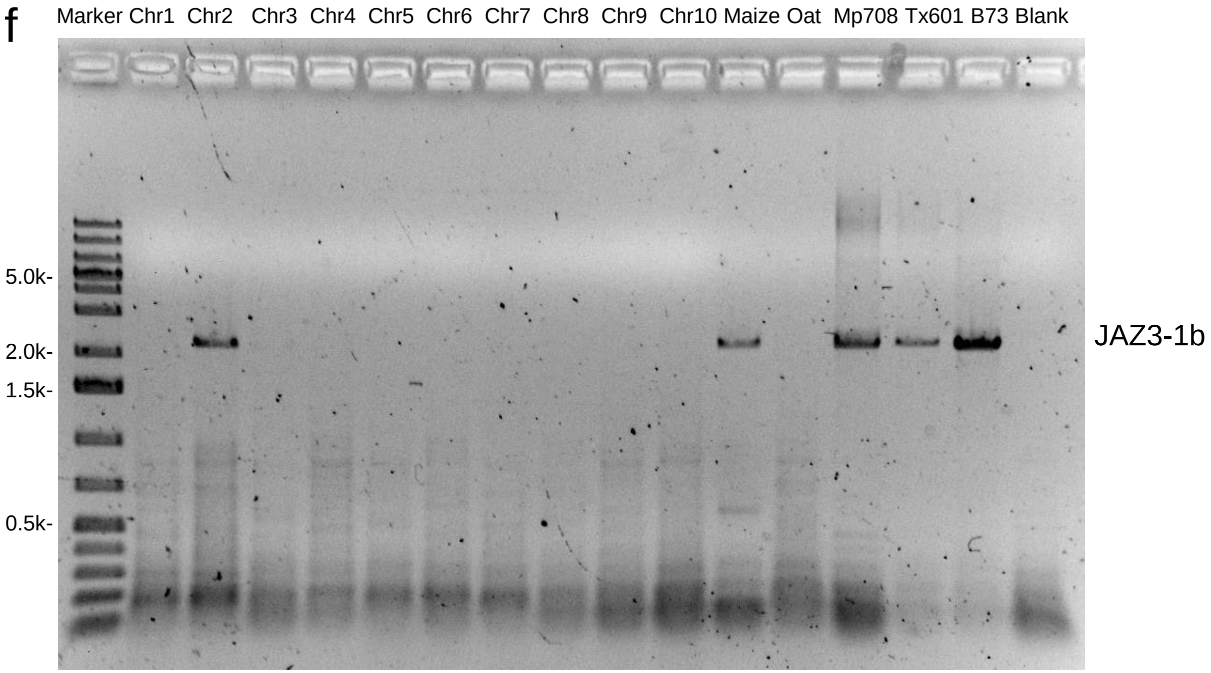

Supplemental Fig. 6 Full-length gels for PCR results with oat-maize addition lines. PCR was performed using specific JAZ primers for gDNA amplification from the oat-maize chromosome addition lines and three maize inbred lines as templates. A total of six homologous JAZ genes (a-f) were tested and labelled on the right panel. The specific PCR bands for each chromosome location were cropped and presented in Fig. 6. Template gDNAs are indicated at the top: lanes marked Chr1-10 indicate oat-maize addition lines containing maize chromosomes 1-10, respectively; lanes marked maize and oat indicate maize donor and oat background, respectively; lanes marked Mp708, Tx601, and B73 indicate three maize inbred lines used in this study. Agarose gel stained with ethidium bromide was shown above
